# ArchesWeatherGen: Skillful and compute-efficient probabilistic weather forecasting with machine learning

**DOI:** 10.1126/sciadv.adx2372

**Published:** 2026-04-22

**Authors:** Guillaume Couairon, Renu Singh, Anastase Charantonis, Christian Lessig, Claire Monteleoni

**Affiliations:** ^1^ INRIA, Paris, France.; ^2^ ECMWF, Bonn, Germany.; ^3^ University of Colorado Boulder, Boulder, CO, USA.

## Abstract

Weather forecasting plays a vital role in today’s society, from agriculture and logistics to predicting the output of renewable energies and preparing for extreme weather events. Deep learning weather forecasting models trained with the next state prediction objective on ERA5 have shown great success compared to numerical global circulation models. Here, we propose a methodology to leverage deterministic weather models in the design of probabilistic weather models, leading to improved performance and reduced computing costs. We design a probabilistic weather model based on flow matching, a modern variant of diffusion models, that is trained to project deterministic weather predictions to the distribution of ERA5 weather states. Our model ArchesWeatherGen surpasses IFS ENS and NeuralGCM on all WeatherBench headline variables (except for NeuralGCM’s geopotential). Our work also aims to democratize the use of generative machine learning models in weather forecasting research.

## Teaser

Residual diffusion models boost the accuracy and efficiency of machine learning–based probabilistic weather forecasting models.

## INTRODUCTION

The field of weather forecasting is undergoing a revolution. Artificial intelligence (AI) models trained on the ERA5 dataset (ECMWF Reanalysis v5) (*1*) can now outperform IFS-HRES (the reference numerical weather prediction model developed by the ECMWF (European Center for Medium-Range Weather Forecasting), in a wide range of scores (*2*–*8*). At the same time, computing costs for making a forecast are orders of magnitude lower. The standard procedure for training these machine learning (ML)–based weather models has been to fit a neural network to predict the next atmospheric state given the current one, with a lead time of 6 to 24 hours. The neural network is then used autoregressively to make forecasts at longer lead times, through time-stepping.

### Architecture

The neural network architectures of ML weather models are typically adopted from the computer vision community, usually by adding priors related to the specificity of processing physical fields from a three-dimensional (3D) spherical atmosphere [local 3D attention for Pangu-Weather (*2*); Fourier Spherical Operators for FourCastNet (*3*); Graph Neural Networks on a spherical mesh for GraphCast (*4*)]. Adding these physical priors usually serves two goals: (i) AI models that have more priors are more interpretable since they more closely relate to their numerical counterparts, which increases trust in these models; (ii) networks with more physical priors generalize better and can reach the same accuracy with fewer parameters and memory footprint.

However, recent work has started questioning this second assumption, showing that architectures with less physical priors can also generalize well with a lower training cost (*5*, *6*, *9*), which could mean that models with more physical priors and fewer parameters are harder to train. These works have adapted vision transformers (*10*), considering ERA5 as latitude/longitude images, and concatenating upper-air weather variables in the channel dimension. This concatenation requires a lot more parameters than 3D processing, so these works still rely on very large neural networks (300M parameters for Stormer, 1.5B for FuXi).

Here, we identify a limitation of 3D local attention, used in the Pangu-Weather architecture. Inside the network, only the features for neighboring pressure levels interact, mimicking the physical principle that air masses only interact locally at short timescales. We find that despite its connection to physics, this prior is computationally suboptimal, and we design a global cross-level attention (CLA)–based interaction layer that improves forecasting skill.

### Limitations of deterministic models

State-of-the-art ML models reach a lower root mean squared error (RMSE) compared to models based on physical simulation, at the cost of producing smoother outputs that lack physical consistency at short (<300 km) spatial scales (*11*). Furthermore, the smoothing compounds over time, as the smoothed outputs are reused as inputs for predicting weather trajectories. Fine-tuning the models for autoregressive rollouts helps to reduce RMSE but increases smoothing even more, resulting in forecasts in between a realistic physical simulation and a smooth ensemble mean. This smoothing phenomenon has several drawbacks: (i) Extreme events are not well represented by the forecast model; (ii) the ensemble of ML predictions does not cover the range of plausible physical scenarios, which is critical to deal with the chaotic nature of weather in the face of initial condition (IC) uncertainty; (iii) smooth outputs that lack physical realism are less useful in some downstream applications that require physical interpretability.

Solving these issues requires sampling from the true probability distribution of possible weather trajectories given our best knowledge of the IC. The distribution of possible weather trajectories can be approximated by combining classical methods with deterministic ML models, for example, generating an ensemble from perturbed ICs (*2*). While the coverage of the resulting trajectories improves, the dynamics of the model given the input is unchanged; this method does not address smoothing as the model was trained to minimize mean squared error (MSE) and not to generate true weather states from the data distribution. We propose solving the smoothing issue by using generative modeling.

### Generative models for weather forecasting

Generative modeling refers to a class of ML techniques that, given a sample dataset, learn to model the underlying probability distribution of the data. This allows one to generate new synthetic data samples without any assumption on the probability distribution. Generative models can learn conditional distributions, i.e., a mapping from labels to distributions. A popular conditional generation task is text-to-image: Given an input text, the goal is to generate an image that is correctly described by that text (*12*–*15*). In the context of weather forecasting, ERA5 is a collection of weather states that can serve as a dataset of samples for generative modeling. Our goal is to generate weather trajectories from a given IC, so the natural framework is to use a weather state as conditional input, and to model the set of possible trajectories given this input. Since ERA5 represents a single historical trajectory, given an IC, there is only a single sample of the conditional distribution, formed by the states following the IC. However, the historical ERA5 dataset from 1979 to present provides enough samples to learn a conditional model with only one sample per IC, meaning that the model learns to approximate the true underlying probabilistic mapping and can generalize to other ICs.

Direct modeling of the distribution of weather trajectories allows us to better cover the range of future weather events compared to using deterministic ML models. Furthermore, well trained generative weather models do not suffer from the smoothing issue of deterministic models, since smoothed outputs do not belong to the true (conditional) data distribution that generative models approximate.

In theory, sampling a distribution over trajectories requires a neural network to predict a full trajectory at once, which is impractical since deterministic ML weather models have been designed to predict individual weather states. However, we can use a Markovian approximation of the true dynamics, where the distribution of the next weather state only depends on the current state or the last two previous states (our default). Learning the generative model then amounts to learning the transition distribution of the next state given the input state(s). Trajectories for longer lead times are then sampled autoregressively by sampling each transition in the trajectory. We choose this approach for ArchesWeatherGen which is also followed in GenCast (*16*) (see Materials and Methods for more details).

While generative models allow one to model the fundamental uncertainty in weather dynamics, large-scale atmospheric processes are still nearly deterministic at short timescales (e.g., 6 to 24 hours), which partly explains the success of deterministic ML models trained for this timescale. The resulting transition distribution for these lead times is narrowly centered around its expectation, reflecting the small uncertainty. Therefore, a large part of the generative training consists of learning this expectation, which is exactly the training objective of deterministic models. This naturally calls for a decomposition of training into two stages: first, learning the deterministic component with the standard RMSE objective, which approximates the distribution mean; second, subtract this deterministic component from the training data and train a generative model on the resulting data, which we call residual data or residuals. This approach was first proposed in the context of super-resolution (*17*) and successfully applied to generative downscaling (*18*). Here, we show that this strategy is computationally much more efficient than generative modeling from scratch on the transition distribution. We also investigate the difficulties that arise from this two-stage approach, which are mainly due to the overfitting of the deterministic model.

We use diffusion-based models (*19*, *20*) as our generative modeling framework, inspired by their huge success in text-to-image generation (*14*, *15*). These models are trained as denoisers: Samples from the data distribution are mixed with Gaussian noise at various signal-to-noise ratios (SNRs), and a neural network is trained to predict the original samples. New samples can be generated by sampling random Gaussian noise and iteratively denoising it with the neural network. The advantage of diffusion-based models compared to generative adversarial networks is believed to be due to the denoising loss function, which provides stable learning and is directly correlated with sample quality (*21*). In this work, we choose a variant of diffusion models called flow matching models (*22*), which we show to perform experimentally better than the original version of diffusion models, denoising diffusion probabilistic models (DDPMs) (*19*, *23*).

### Related work

In this work, we mainly compare ArchesWeatherGen to NeuralGCM (*8*), since its output is available in WeatherBench for evaluation. NeuralGCM is a hybrid physics-ML method, with a dynamical core for numerical simulation and a neural network trained to adjust the simulation to minimize the MSE forecast error.

The most similar work to ours is GenCast (*16*), which is also based on diffusion models for ensemble weather forecasting. In addition to requiring less resources to train (partly because we operate at 1.5° instead of 0.25°), the most notable differences are the architecture [original Transformer (*24*) for GenCast and Swin vision transformer (*25*) for ours], the diffusion paradigm [elucidated diffusion model (*20*) versus Flow matching (*22*)], design choice to increase diversity [ensemble of data assimilation (EDA) perturbations for GenCast, noise scaling, and out-of-distribution (OOD) fine-tuning for us]. In particular, we use flow matching, which is a more recent variant of diffusion, easier to work with, and has shown excellent results in image generation (*21*). Our model also requires much less computational resources for training (about 20 times less including our deterministic model training). We do not compare our model with GenCast since, at the time of writing, the inference output of GenCast is not available for evaluation. Other works have incorporated diffusion models for global weather forecasting, such as SwinRDM (*26*) and Fuxi-extreme (*27*). We did not evaluate these models because they are not publicly available. Diffusion models can also be used to generate more ensemble members from a base set of physically simulated members, e.g., (*28*, *29*).

Diffusion models have also been successful in atmospheric science tasks that require generating stochastic, high-frequency fields. One first example is downscaling (*18*, *30*–*34*). Another natural application of diffusion models in atmospheric sciences is precipitation nowcasting, due to the high stochasticity of the field (*35*–*39*). Other fields that have benefitted from diffusion-based approaches are tropical cyclones forecasting (*40*, *41*) and sea ice modeling (*42*). Last, diffusion-based models are not only competitive in probabilistic modeling, they are also well fitted for data assimilation and have been adapted for this purpose (*43*–*45*).

### Summary of results and contributions

**Fig. 1.**
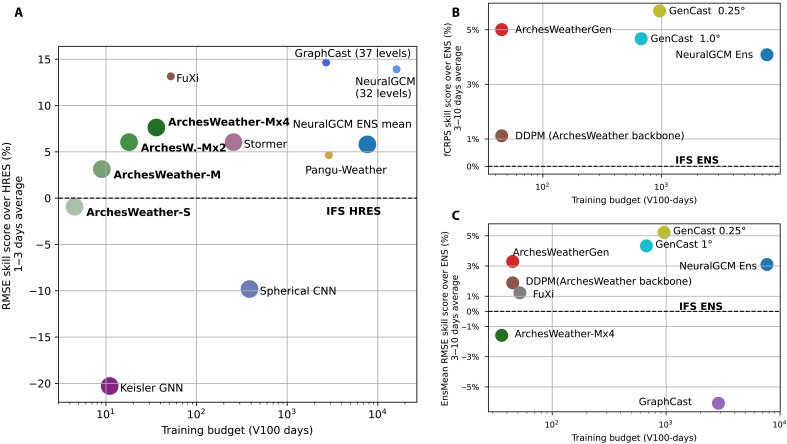
Summary evaluation metrics for the test year 2020 averaged over key upper-air variables (*Z500*, *Q700*, *T850*, *U850*, *and V850*) as a function of training computational budget, comparing ArchesWeather and ArchesWeatherGen to other deterministic and ensemble-based models (higher is better). The computational budget of each model is converted to V100-days, according to the heuristics presented in the Supplementary Materials. (**A**) RMSE skill score over IFS HRES averaged for lead times of 1 to 3 days, comparing ArchesWeather to other state-of-the-art deterministic ML models in WeatherBench2. Circle size indicates training resolution: small circles for 0.25°/0.7°, big circles for 1°/1.4°/1.5°. Compared to the other models trained at a similar resolution, ArchesWeather reaches competitive or better forecasting performance with a much smaller training budget. The two plots on the right display ensemble metric skill scores over IFS ENS, averaged for lead times of 3 to 10 days. (**B**) ArchesWeatherGen reaches better fair CRPS scores (fCRPS; see section S3) than NeuralGCM and the 1.0° GenCast at a much lower computational budget, and our flow matching–based design greatly improves upon the original DDPM diffusion models. (**C**) ArchesWeatherGen also better approximates the ensemble mean than competing methods, including the deterministic model FuXi. GraphCast is shown as reference for a nonensemble-based deterministic model but was not trained to optimize this metric at 3 to 10 days.

1) We show that the 3D local attention in Pangu-Weather is computationally suboptimal, and we design a nonlocal CLA layer that boosts performance. We train our deterministic model ArchesWeather at 1.5° resolution and 24-hour lead time. ArchesWeather achieves competitive RMSE scores with a computational budget orders of magnitude smaller than competing architectures (see Fig. 1). An ensemble average of four models is competitive with the 1.4° NeuralGCM ensemble with 50 members (*8*) for lead times up to 3 days.

2) We show that we can leverage deterministic weather models to train generative weather models with improved performance and reduced computational costs. We use our ArchesWeather models to remove the predictable component from ERA5 and train flow matching models on the residual data, with the same neural network architectures. We show that flow matching models (*22*) perform better than the original version of diffusion models, DDPMs (*19*, *23*). The resulting model ArchesWeatherGen outperforms NeuralGCM on temperature, humidity, and wind components, but not geopotential, as measured by probabilistic forecasting metrics: ensemble mean RMSE, CRPS (continuous ranked probabilistic score), and Brier score.

3) Our work allows for ML-based weather forecasting research with academic resources. ArchesWeather requires only 1 terabyte of data to download (versus 36 times more at 0.25°) and a training budget of 10 V100-days and has an inference cost of 0.25 s for a 24-hour forecast on the same hardware. Our best generative model ArchesWeatherGen requires a computational budget of 45 V100 days (mainly allocated to training four deterministic ArchesWeather models), and inference is longer due to the iterative denoising mechanism of diffusion-based models, which requires 3.5 s per 24-hour forecast on an A100 card.

Figure 1 presents a summary of the WeatherBench metrics of our model, compared to the main other ML weather models. While competing weather models are trained in various settings (different resolution, number of pressure levels, lead time, and compute budget), this figure serves as an overall view of the operating point chosen for each model. As computational efficiency is one of our core focus, we chose to plot the skill of each model (as a relative improvement over a fixed reference model, IFS HRES of IFS ENS) against its training budget. While there is not a unambiguous way to compare computational costs between different GPU (graphics processing unit) cards, the training budgets were taken from the original articles and converted to a single scale (V100-days) according to hardware heuristics presented in section S3.1. ArchesWeather is trained with a computational budget one to two orders of magnitude smaller than competing models yet obtains very competitive results, especially for 3 to 10 days ahead probabilistic metrics.

## RESULTS

We first evaluate our deterministic model ArchesWeather with RMSE, at a lead time of 24 hours as well as longer lead times through autoregressive rollouts. We then showcase some limitations of deterministic models and evaluate our probabilistic model ArchesWeatherGen. All metrics used to evaluate deterministic and probabilistic weather models are presented in the Materials and Methods section and formally defined in the Supplementary Materials (section S3). The design choices of our generative model validated through ablations are discussed in section S4.2 of the Supplementary Materials.

### Evaluating ArchesWeather deterministic models

#### Main results at 24-hour lead time

Table 1 shows RMSE scores of ArchesWeather compared to state-of-the-art ML weather models, including Pangu-Weather and GraphCast, SphericalCNN (*46*), NeuralGCM at 1.4° (50 members ensemble), and Stormer. Data are from WeatherBench 2, except Stormer where we evaluated outputs provided by the authors. Some models do not include surface variables, and hence, their performance cannot be reported.

**Table 1.** Comparison of deterministic ML weather models on RMSE scores for key weather variables at a 24-hour lead time. Cost is the training computational budget in V100-days. Best scores for training resolution coarser than 1° are in underlined bold, second best scores are in bold. N/A, not applicable.

	Res.	Cost	*Z500*	*T850*	*Q700*	*U850*	*V850*	*T2m*	*SP*	*U10m*	*V10m*
	°	days	m^2^/s^2^	K	g/kg	m/s	m/s	K	Pa	m/s	m/s
IFS HRES			42.30	0.625	0.556	1.186	1.206	0.513	60.16	0.833	0.872
Pangu-Weather	0.25	2880	44.31	0.620	0.538	1.166	1.191	0.570	55.14	0.728	0.759
NeuralGCM	0.25	16128	37.94	0.547	0.488	1.050	1.071	N/A	N/A	N/A	N/A
FuXi	0.25	52	40.08	0.548	N/A	1.034	1.055	0.532	49.23	0.660	0.688
GraphCast	0.25	2688	39.78	0.519	0.474	1.000	1.02	0.511	48.72	0.655	0.683
Keisler	1	11	66.87	0.816	0.658	1.584	1.626	N/A	N/A	N/A	N/A
SphericalCNN	1.4	384	54.43	0.738	0.591	1.439	1.471	N/A	N/A	N/A	N/A
Stormer	1.4	256	45.12	**0.607**	0.527	**1.138**	**1.156**	0.570	**53.77**	**0.726**	**0.760**
NeuralGCM ENS	1.4	7680	**43.99**	0.658	0.540	1.239	1.256	N/A	N/A	N/A	N/A
ArchesWeather-S	1.5	9	48.92	0.650	0.542	1.289	1.325	0.562	60.30	0.833	0.873
ArchesWeather-M	1.5	18	44.96	0.615	**0.527**	1.219	1.251	**0.539**	56.22	0.783	0.819
ArchesWeather-Mx4	1.5	36	**41.93**	**0.593**	**0.513**	**1.172**	**1.203**	**0.517**	**52.22**	**0.749**	**0.783**

The ArchesWeather-M base model largely surpasses the SphericalCNN model for upper-air variables, with a training budget of around 9 V100-days, i.e., 40 times smaller. At 24-hour lead time, the ArchesWeather-Mx4 version (a basic ensemble average of four M models) outperforms the 1.4° NeuralGCM ensemble (50 members) on upper-air variables. They perform on par with the original Pangu-Weather(0.25°) and Stormer(1.4°), except for wind variables (*U850*, *V850*, *U10*, and *V10*) where notably Stormer is consistently better. This might be due to the higher training budget (256 V100-days), bigger models, or averaging outputs from 16 model forward passes (more details in section S2 of the Supplementary Materials). Investigating this discrepancy is left for future work.

#### Multistep evaluation

**Fig. 2.**
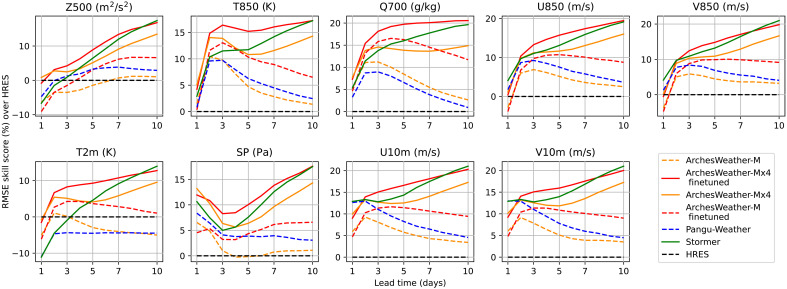
RMSE skill scores of weather models for lead times up to 10 days. Models that do not use ensembling are shown in dotted lines. We can see that ArchesWeather-Mx4 (fine-tuned) surpasses Stormer on all headline metrics for lead times up to 8 days, despite using four times fewer ensemble members (4 versus 16).

We now report scores of deterministic models evaluated at longer lead times through autoregressive rollouts, both with multistep fine-tuning and without. In Fig. 2, we plot the RMSE skill scores (with respect to HRES) of our models ArchesWeather-M (without multistep fine-tuning), ArchesWeather-Mx4 (ensemble of 4 models without multistep fine-tuning), and ArchesWeather-Mx4 fine-tuned (ensemble of four models, each with multistep fine-tuning). The competing methods that we use for reference are Pangu-Weather at 0.25° (closest to our model) and Stormer at 1.4° (best competing method at a similar resolution).

First, we see that the simple strategy of training four independent ArchesWeather models and averaging them allows us to reach better scores than the single models, showing the importance of not relying only on multistep fine-tuning to get good RMSE scores at longer lead times. Second, our ensemble of four fine-tuned models surpasses Stormer on all headline variables for lead times of 2 to 8 days. The better 24-hour RMSE scores for Stormer on wind variables (*U850*, *V850*, *U10m*, and *V10m*) do not translate to better multistep trajectories.

Multistep fine-tuning on autoregressive rollouts allows one to improve the RMSE scores of single trajectories at longer lead times. For ensemble forecasting, only the ensemble mean should reach the minimum RMSE error and not each member of the ensemble, meaning that multistep fine-tuning is not needed. In the remainder of the paper, we use 24-hour ahead underlying deterministic models without multistep fine-tuning.

#### Ablations of the deterministic model

Our main ablation experiment for deterministic models is presented in Table 2, where we compare models without multistep fine-tuning, for two different model sizes, S and M. For a fair comparison between models, we decrease the embedding dimension (by about 5%) when using CLA, so that all types of model have roughly the same parameter count. Our base model without CLA or fine-tuning on recent data [recent past fine-tuning (RPFT)] largely improves performance upon a Pangu-Weather model retrained in the same setting, but the M model is still 11.6% worse than the IFS model, as measured, but the RMSE skill score averaged over all headline variables.

**Table 2.** Ablation of major components of ArchesWeather. Metrics are 500-hPa geopotential and 2m temperature RMSE at 24-hour lead time for different versions of our models (and Pangu-Weather retrained at 1.5°). RMSEss is the relative RMSE improvement over HRES. Models without our proposed CLA module use local 3D attention, except the 2D ArchesWeather that uses traditional 2D attention with vertical information stacked along the embedding dimension.

Model	CLA	RPFT	Swi GLU	Ens.	*Z500*↓	*T2m*↓	RMSEss↑
Pangu-S					66.7	0.84	−30.6
Base improvements					55.1	0.594	−17.1
+CLA	X				50.6	0.567	−9.7
+2007–2018 fine-tuning	X	X			49.3	0.566	−8.6
ArchesWeather-S	X	X	X		48.92	0.562	−5.2
Pangu-M					58.7	0.78	−20.4
Base improvements					51.8	0.572	−11.6
+CLA	X				48.7	0.552	−5.6
+2007–2018 fine-tuning	X	X			48.0	0.551	−5.0
ArchesWeather-M	X	X	X		44.96	0.539	0.66
ArchesWeather-Mx4	X	X	X	X	41.93	0.517	5.3
2D ArchesWeather-M		X	X		53.0	0.575	−8.0

#### Impact of CLA

Upon our baseline model, adding the proposed CLA scheme substantially improves forecasting skill, reducing by half the RMSE difference with the IFS HRES. It represents a +7.4% (absolute) improvement in the RMSE skill score for the S model and +6% for the M model. This is a large improvement: ArchesWeather with 16 layers has better skill than using 32 layers without CLA (−9.7% RMSE skill score versus −11.6%), despite the doubled computational budget. It is also a larger improvement than using an ensemble of four models (a +4.7% improvement), despite the quadrupled computational budget. Furthermore, faster information transfer is more important in models with fewer layers (because there are fewer layers to transfer information), and we observe a larger improvement for the S model than for the M model, which is another indication that CLA fulfills this role.

Given that CLA relaxes the inductive bias for the 3D atmospheric interaction, we also asked whether it could be beneficial to remove the 3D prior entirely by flattening the data along the vertical dimension. This is the setting of the “2D ArchesWeather” in Table 2, whose performance is largely below the best ArchesWeather models despite a much higher parameter count (~850M). This tends to show that the 3D inductive prior is still useful, and the factorized attention better generalizes compared to fully connected matrices along the vertical dimension.

### Comparing generative models to deterministic models

#### Smoothness evaluation

**Fig. 3.**
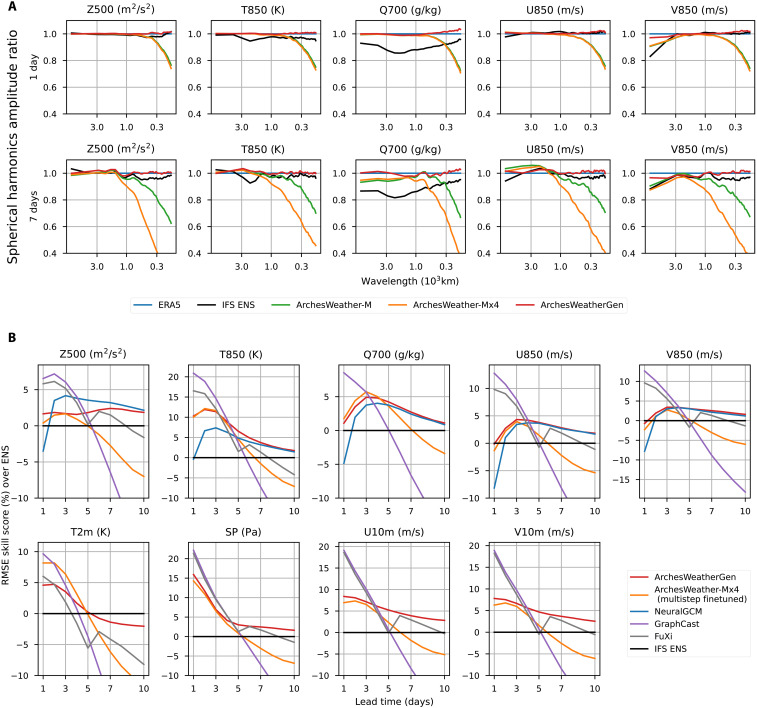
Comparison of deterministic models and generative models on two keys metrics, power spectrum and ensemble mean RMSE. (**A**) Spherical harmonics amplitude ratio for different models. For each model and wavelength, the energy at that wavelength is averaged across samples and divided by the corresponding averaged energy in ERA5. Our model’s power spectrum is much closer to ERA5’s and, on average, better than that of IFS ENS. (**B**) Ensemble mean RMSE skill scores of ArchesWeatherGen, compared to our small deterministic ensemble ArchesWeather-Mx4, NeuralGCM (50 members), and state-of-the-art deterministic models (FuXi and GraphCast). ArchesWeatherGen reaches the best performance on all variables for lead times greater than 4 to 5 days, except *Z500*.

Weather models that produce smooth outputs, like deterministic ML models, are less useful for downstream applications because the forecasts are not physically consistent. While having guarantees for physical consistency remains an open problem, smoothness is a well-defined problem that is measurable with two metrics: activity, which is the SD of the climatology-removed forecast across spatial locations, and power spectra. In Fig. 3, we measure fidelity with respect to the power spectra of ERA5, by averaging the energy of each wavelength across generated forecasts and dividing by the averaged energy at that wavelength in ERA5. We can see that the deterministic models do not have enough energy at short spatial scales, due to smoothing. ArchesWeatherGen’s power spectra are much closer to ERA5’s. It is also better than IFS ENS on *Z500*, *T850*, and *Q700* at a 1-day lead time and better on all variables at a 7-day lead time. We analyze the activity of ArchesWeatherGen across lead times in section S4.2 of the Supplementary Materials.

At a 1-day lead time, we can see that our model has very slightly more energy in the short wavelengths compared to ERA5, especially on *Q700*. However, this error does not accumulate over time, and the 7-day power spectrum ratios are of similar quality compared to the 1-day ratios. Moreover, it is worth noting that ERA5 has the wrong spectrum at these scales.

#### Better approximation of the ensemble mean with generative models

In this section, we evaluate ensemble mean RMSE skill scores of our ArchesWeatherGen model, compared to NeuralGCM, our small deterministic ensemble ArchesWeather-Mx4, and other state-of-the-art deterministic models (FuXi and GraphCast). Although ensemble mean RMSE does not directly measure the ability of models to predict the true distribution over possible futures, it is useful to measure whether predicted trajectories have the correct expectation or whether they are biased. In Fig. 3, we report the ensemble mean RMSE of our models compared to competing methods, including deterministic models. In this plot only, we do not correct for statistical bias for a more fair comparison between ensemble and deterministic models, since members generated with deterministic methods cannot necessarily be scaled easily (for instance, our ArchesWeather-Mx4 model would require to retrain models with other random seeds to generate more than four members). Using this noncorrected metric does favor methods that are able to produce many different ensemble members, which is a desired behavior.

Our ensemble of four deterministic ArchesWeather-Mx4 models has better scores than GraphCast, which shows the importance of using multiple members to approximate the ensemble mean. FuXi reaches better scores because it was trained to directly approximate the ensemble mean after 5 days, unlike GraphCast or ArchesWeather with multistep fine-tuning. Ensemble methods (NeuralGCM and ArchesWeatherGen) better capture the ensemble mean compared to FuXi, which shows the advantage of using sampling ensemble members rather than predicting the ensemble mean with a neural network. Last, ArchesWeatherGen reaches better ensemble mean RMSE scores than NeuralGCM for upper-air variables (NeuralGCM does not predict surface variables), except for geopotential. We make the hypothesis that this could be due to the nature of the *Z500* field, which is smoother and likely very well modeled by NeuralGCM’s dynamical core. The difference between ML-only models and hybrid models is discussed more thoroughly in the Discussion section. Last, we caution that there is a slight penalty for NeuralGCM which is due to the regridding of NeuralGCM’s outputs at 1.4° to the 1.5° evaluation grid, but we were not able to assess its importance, having only access to the 1.5° outputs. It could partly explain NeuralGCM’s lower scores at a 24-hour lead time; however, the regridding penalty’s relative contribution to skill scores should sharply decrease as lead time increases.

### Quantitative evaluation of generative models

In this section, we quantitatively evaluate our main model ArchesWeatherGen, compared to other weather ensemble models: IFS ENS, NeuralGCM, and GenCast (at 0.25° and 1.0° resolution). We also evaluate a baseline version called ArchesWeather-DDPM, which uses our neural network architecture as the backbone for DDPM diffusion models, without flow matching, OOD fine-tuning, or noise scaling. All models are evaluated using an ensemble size of 50 members.

#### Summary of metrics

**Fig. 4.**
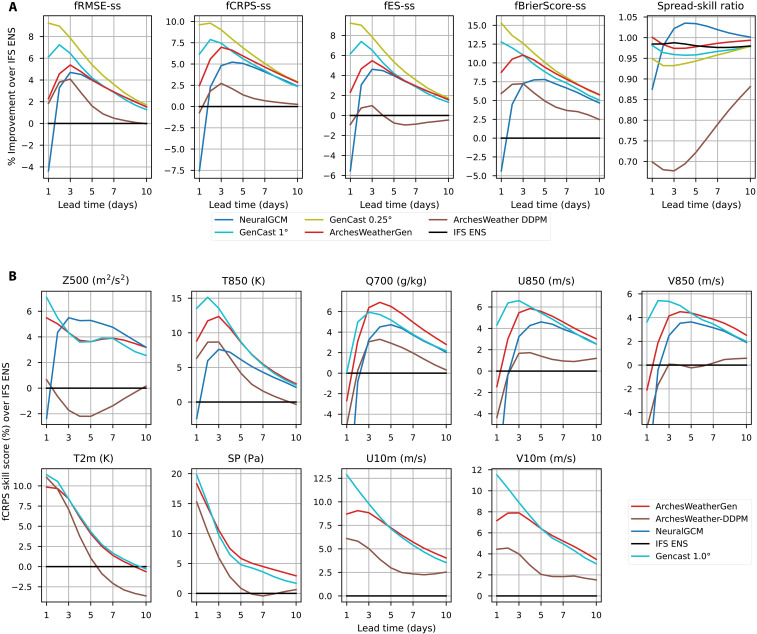
Evaluation of ArchesWeatherGen compared to other weather ensemble models. (**A**) Headline scores are shown as relative improvement with respect to IFS ENS, for RMSE, CRPS, energy score, and Brier score (1% tails), and averaged over our headline upper-air variables: *Z500*, *T850*, *Q700*, *U850*, and *V850*. ArchesWeatherGen surpasses NeuralGCM, has worse scores than the 1.0° GenCast for lead times smaller than 3 days but slightly better scores after 4 days, and converges to the performance of the 0.25° GenCast at 9 to 10 days. ArchesWeatherGen has similar spread-skill ratio as IFS ENS. (**B**) fCRPS skill scores for headline physical variables in WeatherBench, comparing our models to NeuralGCM and IFS ENS. ArchesWeatherGen surpasses IFS ENS, ArchesWeather-DDPM, and has better scores than NeuralGCM, except on geopotential for lead times of 3 to 10 days. Similarly to the global picture above, ArchesWeatherGen has lower skill than the 1° GenCast for lead times before 3 days but slightly better after 4 days.

In Fig. 4, comparison between the different models is based on the key metrics for ensemble evaluation: ensemble mean RMSE (RMSE for short), CRPS, energy score, Brier score (average of scores with thresholds of 1 and 99%) and spread-skill ratio. All metrics are shown as relative improvement over IFS ENS. To be able to compare our averaged skill scores to NeuralGCM, in Fig. 4 only, we average skill scores only on the upper-air headline variables of WeatherBench (*Z500*, *Q700*, *T850*, *U850*, and *V850*).

We observe that ArchesWeatherGen has better scores than IFS ENS and even NeuralGCM for all lead times up to 10 days. Our model reaches ensemble mean RMSE scores similar to those of NeuralGCM for lead times greater than 5 days, which we had also observed in Fig. 3. Compared to GenCast at 1° resolution, ArchesWeatherGen has less skill for 1- to 3-day lead time but then obtains similar or better scores for a lead time of 4 to 10 days. We also observe that ArchesWeatherGen is competitive with the 0.25° GenCast at lead times of 9 to 10 days, which is remarkable given the computational budget 20 times higher and the much higher training resolution.

ArchesWeatherGen is very slightly underdispersive, similar to IFS ENS. Compared to Arches Weather-DDPM, we first see that our model brings an important improvement over the original DDPM diffusion models, with relative CRPS scores roughly 4% better for all lead times. The DDPM baseline is very underdispersive, with a spread-skill ratio of 0.7 for 24 hours, increasing to 0.88 at a 10-day lead time, which flow matching and our other proposed improvements address.

#### Per-variable CRPS skill scores

While in the last section we have shown metrics averaged over headline variables, we now investigate scores for each physical variable independently. We focus here on the CRPS scores, which measure the quality of marginal distributions as well as requiring the autoregressive models to make physically consistent predictions to have good scores at longer lead times. We report these scores across lead times in Fig. 4, comparing ArchesWeatherGen with ArchesWeather-DDPM, NeuralGCM, and the 1.0° GenCast.

ArchesWeatherGen reaches better CRPS skill scores than IFS ENS across all headline variables and lead times, on average by 5.3%. It is slightly worse only for *T2m* at lead times longer than 9 days. It improves upon NeuralGCM on all headline variables and lead times, except geopotential for lead times of 3 to 10 days. This advantage for NeuralGCM, which we have already observed on ensemble mean RMSE, might again be due to the dynamical core modeling, which is better suited for capturing the evolution of geopotential height. Compared to DDPM, using our model yields notable CRPS improvements on all variables, generally by 2 to 4% relative points across lead times and up to 5 to 6% for geopotential. As geopotential is the variable for which overfitting of the underlying deterministic model is the strongest, we believe that this larger gain for geopotential demonstrates that our proposed improvements to tackle overfitting are working.

The per-variable CRPS scores of ArchesWeatherGen compared to GenCast at 1° resolution reveal the same pattern as for the global picture: worse score for lead times of 1 to 3 days (especially on wind variables) and better or similar scores for lead times of 4 to 10 days. We make the hypothesis that this gap is due to the higher training resolution (twice more effective training data) and perhaps the 12-hour lead time used for training compared to 24 hours in our case, which might better resolve small timescale atmospheric processes.

### Qualitative analysis on Hurricane Teddy

**Fig. 5.**
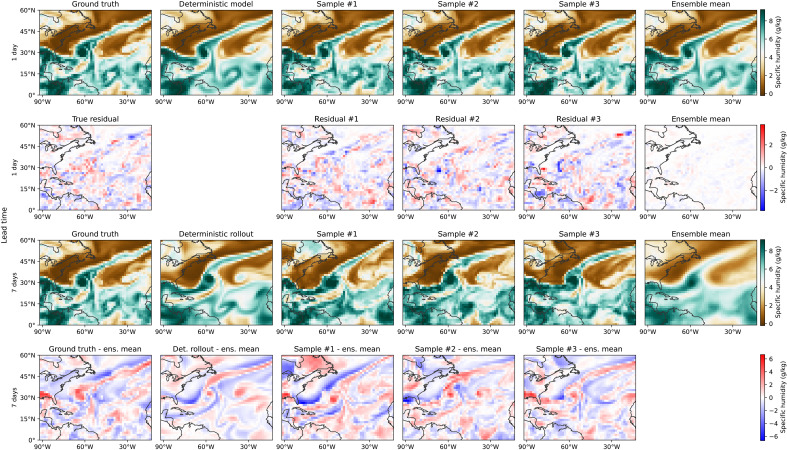
Visualization of 700-hPa specific humidity predictions made by our generative weather model on Hurricane Teddy at 12 p.m. on the 21st of September 2020. Predictions are made from 1 day before this date (two first rows) and 7 days before this date (two last rows). On the second row, we visualize the residuals generated by our flow matching model, which are added to the 1-day deterministic prediction to recover the 1-day ensemble predictions. The true residual is computed by subtracting the deterministic prediction to the ground truth state 24 hours after initialization. One the last row, we visualize the difference between samples and the ensemble mean, computed from a 50-members ensemble. The deterministic predictions are made by our deterministic model and are blurrier than the ground truth.

In Fig. 5, we show humidity samples generated by our model for Hurricane Teddy on 21 September 2020. Hurricane Teddy was the fifth-largest Atlantic hurricane by diameter of gale force winds recorded. It produced large swells along the coast of the eastern United States and Atlantic Canada. It resulted in massive damages over the island of Bermuda on the 21st of September when the storm suddenly intensified. In the first line of the figure, we present the cyclone as reanalyzed in ERA5 and compare it with the 1-day ahead forecast of the deterministic model (ArchesWeather), some samples of the 50-member generated ensemble of ArchesWeatherGen, and their ensemble mean. As expected, the ensemble mean is extremely close to the deterministic prediction. However, we can observe some small-scale variations on the individual samples, such as the convection plume splitting in sample two, and slight displacements of the small-scale structures, corresponding to slight variations in the trajectory of the hurricane. These can be observed in the shape of the errors, generally corresponding to small differences in the advection of water masses. In all these cases, the forecast passes very close to Bermuda.

If we examine the 7-day rollouts of both the deterministic model and the generative model, all models predict the intensification from a much weaker version of the cyclone on 14 September (their IC). We can notice a much higher variance in the generated samples, corresponding to different possible trajectories. This is reflected in the smoothness of the ensemble mean, corresponding to averaging different larger-scale structures.

Individual trajectories obtained with the generative approach contain sharp features compared to the deterministic rollout, which becomes smooth at a 7-day lead time. This sample sharpness is further illustrated by the clear large-scale structures that emerge when visualizing the differences between the individual members and the ensemble mean, seen on the fourth line of the figure. This corresponds to different trajectories that the hurricane could have followed and variations in its intensity. We can also observe the formation or dissipation of a secondary, weaker, cyclonic structure eastward of Hurricane Teddy in the 7-day rollouts. Multiple of the generated trajectories remain close to the actual path of the hurricane. Twenty-five more samples can be visualized in fig. S9 of the Supplementary Materials.

## DISCUSSION

### Learning spherical data with transformers

Our data source is the classical equirectangular projection of the ERA5 dataset, which presents some modeling challenges: Physical phenomena are distorted near the poles, requiring faster information exchange in the neural network. We tried accounting for the sphere topology in two ways: First, we experimented with a shifting mechanism in swin transformers that connects some parts of the northern data that are otherwise very distant in the latitude-longitude representation. This did not improve the forecasting error since at 1.5°, information exchange in attention windows covers long distances, and any two regions on Earth can exchange information throughout the model. However, in ArchesWeather, as in Pangu-Weather, we do connect the left-most (180°W) and right-most (180°E) image parts at every layer, making our data and network effectively cylindrical.

Second, we tested weather forecasting on a cubed Earth projection, with a stack of six faces and attention windows that can exchange information across neighboring faces. While this seems promising, we did not pursue further due to two hurdles: (i) The 1.5° data are downsampled from the 0.25° equirectangular projection, which results in the 1.5° data having some high frequencies in the high latitudes than cannot be obtained from projecting a 1.5° cubed Earth map. (ii) In WeatherBench, the reference data for computing the forecast error is the equirectangular map at 1.5°. Training a model on another representation, like cubed Earth, exposes the model to an additional reprojection error when going back to the latitude-longitude format for computing metrics in WeatherBench. Therefore, the cubed Earth neural network needs to be trained with an MSE loss on the equirectangular map, requiring a differentiable projection after the transformer layers to convert the cubed Earth format. This differentiable projection would allow the neural network to compensate for the reprojection error. These two problems are not major blockers, and we believe that, at 0.25°, a differentiable projection back to the equirectangular map, using transformers on a cubed Earth representation, could potentially bring improvements, as it did for convolutional neural networks (*47*).

### Conceptual difference between ML-only approach and hybrid physics-ML approach

In this work, we have mainly compared our model to NeuralGCM, whose predictions are based on a physics-based simulation with a dynamical core. We have shown that this hybrid approach performs better on the geopotential field, which is smoother and well resolved by the dynamical core. The other fields (temperature, humidity, and wind components) are higher frequency fields, and the grid-scale physical phenomena are less well resolved at 1.5° resolution. Since the 24-hour dynamics at this resolution are complex and hard to resolve via a dynamical core, our ML-only approach directly learns them from data, which is also computationally much more efficient than dynamical simulation since the 24-hour target state is directly predicted, without time-stepping. However, the dynamical simulation puts more constraints on the predicted forecasts and acts as a strong physical prior. This should help improve generalization (*8*), which could be worth the additional computational cost. Investigating this compute-generalization trade-off could be an interesting study.

### Overfitting and underdispersion

We have presented a methodology for training diffusion-based generative models, based on removing the deterministic component with an ensemble of deterministic models trained with the RMSE. Overfitting of the base deterministic model gives smaller residual data, on which the generative model is trained. With a similar spread between train and test for the generative models and worse ensemble mean RMSE on the test set compared to the train set, the test spread-skill ratio is lower than on the train set, resulting in underdispersion. To reduce this effect, we fine-tune our model on data from 2019, on which the deterministic model is not trained. However, more gains could potentially be obtained by allocating more training data for this fine-tuning stage, which means training the deterministic model on fewer data. However, any such gains from fine-tuning the generative on more data could be more than compensated for by a deterministic model less skilled, since trained on less data. Determining the optimal data split is left for future work.

### Non-Gaussian residual data

By removing the expectation component learned by deterministic models, the data might not follow a Gaussian distribution anymore, which we have confirmed experimentally: The tails are much heavier than for normally distributed data, with frequent outliers that have a high value after unit variance rescaling. This might make diffusion modeling harder (*48*). We have tried to map these data to a Gaussian distribution via a contraction mapping f (e.g., square root or log), modeling f (x), and then applying $f^{-1}$ to map back to a real sample. Although this improved metrics for the first few autoregressive steps, mapping back with $f^{-1}$ gave us instabilities in the rollouts. We did not pursue this further and leave this for future work.

### Multistep fine-tuning

We have approached the problem of generating consistent trajectories by modeling the transition function and then composing it autoregressively, which relies on a Markovian assumption of the underlying data/physical phenomenon. While this assumption seems to hold well in practice, its limitations could theoretically be addressed by fine-tuning the model on autoregressive rollouts. Fine-tuning could also help to correct small modeling defects in the one-step transition function and improve multistep predictions. Although this strategy is beneficial for deterministic models, we found in fig. S6 that the use of multistep fine-tuned models as base models in our framework is harmful, because it slightly degrades the quality of the transition model. However, the gap between the two methods reduces to 0 as lead time increases, meaning that either (i) the bias introduced by multistep fine-tuning is less and less important over time or (ii) multistep fine-tuning slightly helps for generative rollouts but it is more than compensated by the worse model metrics at 24 hours, which could be addressed with a better multistep fine-tuning procedure. We leave investigating this for future work.

Another possibility is to fine-tune the generative model itself from its rollouts. However, this requires us to fine-tune models through many diffusion sampling steps, whose naive implementation is impracticable.

### Diffusion modeling and IC perturbation

In this work, we do not consider perturbations of the IC to generate weather trajectories. In theory, even if there is some uncertainty in the true weather state $x_{t}$ that affects the weather trajectories, the learned transition distribution $p\left(x_{t+\delta }|x_{t}\right)$ should take this uncertainty into account, unlike traditional physical models. In practice, the GenCast model uses diffusion modeling, as well as IC perturbation derived from the ERA5 EDA (*49*). We show that, without perturbation, the diffusion model is underdispersive. Although we have also observed underdispersiveness for our model, we have proposed other solutions that are simpler to implement compared to IC perturbation and solve the underdispersion issue. This might indicate that IC perturbation is not needed to generate weather trajectories from diffusion models with the correct dispersion. However, using an ensemble of IC conditions does provide more information about the uncertainties of weather conditions and can improve the skill of the model. The impact of using EDA perturbations on skill was evaluated by the authors of GenCast (*49*), and the overall effect was found to be very limited, with a relative improvement of less that 1%. Therefore, we found the skill difference not worth the added complexity of using EDA perturbations that comes with nontrivial design choices.

### Comparing ML weather models and physics-based weather models at different resolutions

Increasing the resolution of a numerical weather simulation allows one to resolve more physical processes, and this generally improves the forecasting skill for weather predictions. Higher-resolution simulations are beneficial at short lead times (e.g., up to a couple of days), and it is also generally the case that improvements at short lead times cascade into skill improvements at longer lead times (up to 10 to 15 days). It was therefore natural to expect the same for ML weather models. In this study, we found that while ArchesWeather trained at a 1.5° resolution is worse than the GenCast model trained at 0.25° for small lead times, it reaches very similar scores at lead times of 9 to 10 days. Our results demonstrate that one can train on coarser scale data without substantial degradation of prediction performance on 9- to 10-day timescales. We believe that this is possible because the 1.5° ERA5 data (downsampled from 0.25°) represent the historical atmospheric states more faithfully than a 1.5° physics-based numerical simulation. It suggests that features at finer resolution than 1.5^о^ do not help to reduce the average forecasting error at 10- to 15-day lead time; however, those finer features could still help better resolve events in the tails of the distribution.

### Perspectives for future work

We have presented ArchesWeather and ArchesWeatherGen, two ML models for weather prediction that operate at 1.5° resolution. ArchesWeather is a deterministic model trained with a 24-hour lead time and has very good skill thanks to various neural network architecture innovations. ArchesWeatherGen is a flow matching model with the same backbone architecture, trained to project predictions from ArchesWeather to the full distribution of ERA5 weather states, enabling probabilistic forecasting. We find that ArchesWeatherGen surpasses IFS ENS and even NeuralGCM on most physical variables, as measured by probabilistic ensemble metrics, including ensemble mean RMSE, CRPS, and Brier score. We have found that a main obstacle to unlocking the full potential of residual modeling is overfitting of the underlying deterministic model. Fine-tuning the model on data that the deterministic model has not been trained on and scaling the initial noise of the sampling process were effective strategies to tackle this problem. Our best model can be trained with a computational budget of 23 A100 days, paving the way for more accessible research on ML weather forecasting.

Our generative model not only provides accurate future weather trajectories but is also naturally suited for data assimilation. Diffusion-based models (including flow matching models) can be used to predict weather states for which we have measurements and want to sample the conditional distribution with respect to these measurements, for example, with score-based data assimilation (*44*, *45*). Operating at 1.5°, our models are, however, less suited for applications that require a better resolution, like cyclone tracking or regional forecasting. The output of our models could potentially be downscaled to a finer resolution, which we leave for future work.

## MATERIALS AND METHODS

We first present our problem setup and proposed neural network architecture; we then cover our methodology for training and evaluating both deterministic and generative weather models. Our objective is to predict the evolution of weather variables in the ERA5 dataset (*1*) regridded to 1.5° resolution, which is the standard used for evaluation at the World Meteorological Organization. Following the standard in Weatherbench 2 (*50*), we use 1979 to 2018 as the training period and test our models at 00/12 UTC for each day of 2020. For our generative model, we sometimes use 2019 as a fine-tuning period. We consider standard hyperparameters for training and hence do not use a validation set.

A weather state at time t is noted $x_{t}\in {\mathbb{R}}^{n}$ and consists of six upper-air variables (temperature, geopotential, specific humidity, and wind components U, V and W) sampled on a latitude-longitude grid at 13 pressure levels and four surface variables (2m temperature, mean sea level pressure, and 10-m wind U and V). $D={\left(x_{t}\right)}_{t\in T}$ is the historical trajectory of weather variables, sampled every 6 hours. Given an input state $x_{t}$, we aim to predict the future trajectory ${\left(x_{t+\mathrm{k\delta}}\right)}_{1\leq k\leq K}$, where δ is the lead time, which is set to δ=24 hours for the remainder of the paper. Both our deterministic and generative weather models require one to map an input state $x_{t}$ to an estimation of $x_{t+\delta }$. In the next section, we present our design of neural architecture for this task.

### Architecture

Our neural network architecture is a 3D Swin U-Net transformer (*25*, *51*) with the Earth-specific positional bias, largely inspired by the Pangu-Weather architecture (*2*) which first proposed this design choice. The surface and upper-air variables are first embedded into a single tensor of size (d,Z,H,W) where d is the embedding dimension, Z is the vertical dimension, and H and W are the latitude and longitude dimensions. This tensor is then processed by the U-Net transformer and is projected back to surface and upper-air variables at the end.

#### Local 3D attention in Pangu-Weather

The attention scheme in a Swin layer (*51*) consists of splitting the input tensor into nonoverlapping windows, where a self-attention layer processes data in each window independently. The input tensor is then shifted by half a window to compute the next self-attention layer, allowing interaction between the different attention windows. In Pangu-Weather, the input tensors are split into 3D windows of size $\left(Z_{\text{window}}=2,H_{\text{window}}=6,W_{\text{window}}=12\right)$: Hence, along the vertical Z dimension, only the features for neighboring pressure levels interact, mimicking the physical principle that air masses only interact locally at short timescales. This inductive prior is meant to have the neural network roughly reproduce physical interaction phenomena and reduce the number of parameters needed.

From a computational perspective, this prior is a limitation since computations for similar phenomena happening at different atmospheric layers are performed independently in parallel. Global vertical interaction would allow sharing of such computations and allocating resources more efficiently. Computations for complex variables can also be spread across levels faster, to reach lower error. Last, from a physical perspective, having vertical interaction can allow us to detect the vertical profile of the atmosphere and to adjust computations accordingly.

Before presenting our proposed solution, we mention two other potential methods and their caveats. First, one could increase the attention window size, e.g., to (4, 6, 12) instead of (2, 6, 12), to accelerate exchange of information along the vertical dimension, but this decreases inference speed due to the quadratic cost of attention in the sequence length. Second, some works use a more standard 2D transformer (*5*, *6*) and stack variables across pressure levels in a single vector at each spatial position. This comes at the cost of an increased parameter count: With Z pressure levels (after embedding), the linear and attention layers need $O\left(d^{2}Z^{2}\right)$ parameters, with d being the embedding dimension for a single pressure level. As a result, Stormer uses a ViT-L with 300M parameters (Vision Transformer, L size) and FuXi uses a SwinV2 architecture with 1.5B parameters.

#### Improving attention with CLA

**Fig. 6.**
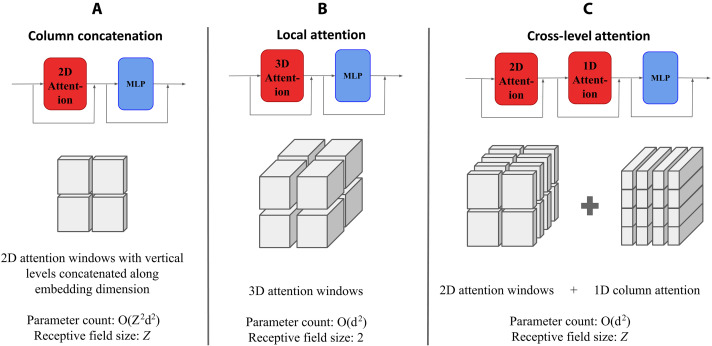
**Visual Comparison of attention schemes for 3D data.** These attention schemes are used in FuXi/Stormer (**A**), Pangu-Weather (**B**) versus ours (**C**). Our design has the highest receptive field size without requiring a number of parameters scaling quadratically with respect to the number of layers *Z*.

We propose making all vertical features interact by adding a column-wise attention mechanism which we call CLA, which processes data along the vertical dimension of the tensor only. Considering the column data as a sequence of size Z, the number of parameters in this attention module is $O\left(d^{2}\right)$ and does not depend on Z. We also remove the vertical interaction from the original implementation by using horizontal attention windows of shape (1, 6, 12), which reduces the cost of attention. The resulting attention scheme shares similarities with axial attention (*52*) with a decomposition of attention into two parts: column-wise attention and local horizontal 2D attention. We note that this added vertical interaction increases the representation power of the neural network with very few additional parameters, which allows us to model a broader class of functions compared to the local attention and better fit the training data. See Fig. 6 for an illustration of our proposed attention scheme, compared to other attention methods. Axial attention has also been used in MetNet-3 (*53*) and SEEDS (*28*).

#### Additional design choices

The standard for vision transformers used in dense tasks, like weather forecasting, is to use a strided deconvolution layer for the final projection (*2*, *5*). However, this layer generally produces unphysical artifacts. We solve the issue by initializing the deconvolution layer with the ICNR scheme (*54*). We also use the SwiGLU activation function (*55*) in the network’s MLP layers instead of ReLU. SwiGLU is the de facto standard for modern large language models (*56*) and brings a substantial improvement to the model’s performance (reducing negative log-likelihood without increasing the number of parameters). Last, following GraphCast, we provide additional information to the model (hour and month of desired forecast) with adaptive layer normalization (*57*).

### Training and evaluating deterministic weather models

Deterministic ML models, which we note $f_{\theta }$ are trained by adjusting their parameters θ to minimize the weighted MSE between the predicted state $f_{\theta }\left(x_{t}\right)$ and the ground truth state $x_{t+\delta }$

$$L\left(f_{\theta }\left(x_{t}\right),x_{t+\delta }\right)=E_{t\in D}∥f_{\theta }\left(x_{t}\right)-x_{t+\delta }{∥}_{S}^{2}$$ (1)

$∥x{∥}_{S}^{2}$ is a weighted mean of squared values in the tensor x. The weighting comes from two sources: First, latitude weighting accounts for the area distortion introduced by the latitude/longitude representation of spherical data. Second, we use the same coefficients as GraphCast (*4*) to sum the contribution of different physical variables. In particular, we use a coefficient proportional to air density for upper-air variables and specific coefficients for surface variables: 0.1 for mean sea level pressure and 10-meter wind components and 1 for 2m temperature. Two-meter temperature is assigned a much higher coefficient due to its importance as a predicted variable.

#### Detailed protocol for training ArchesWeather

We train two versions of our architecture: ArchesWeather-S (16 transformer layers, 44M parameters) and ArchesWeather-M (32 layers, 84M parameters). The training protocol is as follows:

1) Phase 1: Train models with the MSE loss for 250,000 gradient updates steps.

2) Phase 2: Fine-tune models on recent data (2007–2018) for 50,000 gradient update steps. We find that forecasting models have a higher error in the first half of the training period 1979–2018 (see fig. S1 in section S4.1 of the Supplementary Materials), which we believe that is due to ERA5 being less constrained in the past because of a lack of observation data (*1*). We found that fine-tuning on recent data helps to overcome this distribution shift, a process which we call RPFT.

3) Phase 3 (optional): Fine-tuning on autoregressive rollouts for 20,000 steps. Models optimized for a single lead time of 6 to 24 hours are suboptimal in terms of RMSE scores when used autoregressively for longer lead time predictions. A standard procedure to improve RMSE scores at longer lead times is to fine-tune the model on autoregressive rollouts, summing the MSE losses at each rollout step before backpropagation (*4*–*6*). We fine-tune our model on autoregressive rollouts of length 2 days for 8000 steps, 3 days for 8000 steps, and 4 days for 4000 steps, with gradients from the full trajectory, and call this procedure multistep fine-tuning.

Training the ArchesWeather-M model takes around 2.5 days on two A100 GPUs. Last, we train a small ensemble of our M models, called ArchesWeather-Mx4, by independently training multiple models with different random seeds and averaging their outputs at inference time. This helps remove modeling errors due to initialization and better approximates the true ensemble mean (*58*). Since a better ensemble mean prediction is desirable in residual modeling, we use ArchesWeather-Mx4 as our default underlying deterministic model in our generative training pipeline.

#### Limitations of deterministic models

Because of the MSE loss, deterministic models produce an approximation of the mean of all possible future next states $E\left[x_{t+\delta }|x_{t}\right]$, which is a weather state that is smooth and not physically consistent due to uncertainty. This raises several problems: First, computing reliable statistics of extreme events from forecasts requires a faithful reproduction of the true distribution; second, smooth forecasts tend to misrepresent the full weather variability, even when using IC perturbation to get a set of possible trajectories. Third, the multistep dynamics of ML weather models are not well represented. ML models have never seen smooth states as input, yet they are expected to be used autoregressively as emulators. Empirically, autoregressive rollouts do work, but models are often fine-tuned with multistep rollouts to improve RMSE scores at longer lead times, which increases smoothing even more due to minimizing MSE scores for a more distant and uncertain future.

The trade-off between minimizing RMSE and producing physically consistent weather states (*59*) implies that RMSE of individual forecasts is not the correct metric to train or evaluate weather models. One can only get the best of both worlds by producing a set of weather trajectories all physically consistent and only expecting the mean of these predicted trajectories to reach the lowest possible MSE. To address probabilistic weather generation, we use flow matching models, a modern variant of diffusion models.

### Training flow matching weather models

**Fig. 7.**
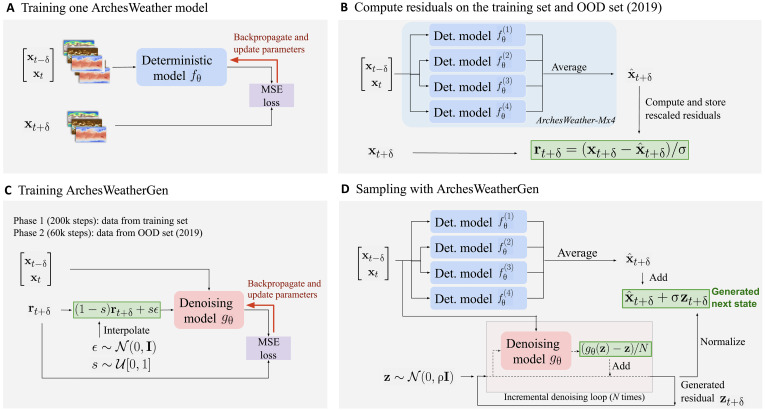
Main overview of the training pipeline of ArchesWeatherGen. (**A**) We train four ArchesWeather models by training neural networks to predict the next state with MSE loss. (**B**) We compute normalized residuals on the training and OOD sets, using ArchesWeather-Mx4 which is the average of four ArchesWeather models. (**C**) We train our flow matching model on the residual data r, i.e., we train a neural network to map residuals corrupted with Gaussian noise to their uncorrupted version. (**D**) We sample ArchesWeatherGen by predicting the mean component with ArchesWeather-Mx4 and then iteratively denoise Gaussian noise to generate a residual sample, which is normalized and added to the mean to recover a complete sample of $x_{t+\delta }$. The sampling process is then used autoregressively starting again with t=t+δ to generate multistep trajectories. Here, we consider δ=24 hours and = 1.05.

We now discuss the background and motivation for training ArchesWeatherGen. Before going into the details, we present an overview of our methodology in Fig. 7.

Our goal is to sample weather trajectories given an IC by learning the transition distribution: We train a model to sample the distribution of next state $x_{t+\delta }$ given the current state $x_{t}$ and previous state $x_{t-\delta }$ and then compose our model autoregressively. This corresponds to the Markovian approximation

$$p\left({\left(x_{t+\mathrm{k\delta}}\right)}_{1\leq k\leq K}|x_{t},x_{t-\delta }\right)≃\prod_{1\leq k\leq K}p\left(x_{t+\mathrm{k\delta}}|x_{t+\left(k-1\right)\delta },x_{t+\left(k-2\right)\delta }\right)$$ (2)

In the remainder of the paper, we will write the transition function $p\left(x_{t+\delta }|x_{t}\right)$ and implicitly consider the dependency on $x_{t-\delta }$ to simplify notations and presentation.

#### Diffusion models and flow matching models

For a general introduction to diffusion-based models (broadly defined), we refer the reader to (*19*, *23*, *60*–*62*). Here, we present concisely how these models are trained and used to model a transition distribution $p\left(x_{t+\delta }|x_{t}\right)$. Diffusion-based models are trained to denoise samples from the data distribution that have been corrupted with Gaussian noise at all SNRs. For the original implementation of diffusion models, DDPMs, samples are mixed with Gaussian noise ϵ∼N(0,I) using the function $z_{t+\delta }\left(s\right)=\sqrt{\alpha_{s}}x_{t+\delta }+\sqrt{1-\alpha_{s}}\epsilon$. Here, s is the diffusion timestep, going from 0 to 1, which controls the level of noise added to the sample through the noise schedule $\alpha_{s}$. With $\alpha_{0}=1$, no noise is added, and $z_{t+\delta }\left(0\right)$ is a sample from the data distribution; with $\alpha_{1}=0$, the sample is completely corrupted, and $z_{s+\delta }\left(1\right)$ is a sample from the Gaussian distribution. Given samples corrupted with all scales of noise levels, diffusion model training optimizes a neural network $g_{\theta }$ to predict the original samples using the loss function

$$L=E_{x_{t}\in D,s∼U\left[0,1\right]}\;\gamma_{s}∥g_{\theta }\left(x_{t},z_{t+\delta }\left(s\right)\right)-x_{t+\delta }{∥}_{S}^{2}$$ (3)

where $x_{t}$ is given as input to the diffusion model to model the conditional distribution $p\left(x_{t+\delta }|x_{t}\right)$ and not just the unconditional distribution $p\left(x_{t+\delta }\right)$.

The coefficients $\gamma_{s}$ control the relative contributions of the denoising objective at each noise level $\alpha_{s}$. Another common training objective is to predict the noise ϵ added to samples (*19*), which is equivalent to predicting samples (since we can find the original sample $x_{t+\delta }$ from the predicted noise ϵ), up to a change of coefficients $\gamma_{s}$. While sample prediction is easy at high SNRs and difficult at low SNR, noise prediction is the exact opposite: easy at high SNR and hard at low SNR. To rebalance the difficulty of the learning objective across all noise levels, we follow the coefficients $\gamma_{s}=\sqrt{\text{SNR}\left(s\right)}=\left(1-s\right)/s$ in (*63*) that provide a more stable loss.

Flow matching models (*22*) are a variant of diffusion models trained with a linear interpolating scheme for the noising function: $z_{t+\delta }\left(s\right)=\left(1-s\right)x_{t+\delta }+\mathrm{s\epsilon}$ for s∈[0,1]. They are usually trained to predict $x_{t+\delta }-\epsilon$ instead of $x_{t+\delta }$, which corresponds to the flow velocity between the noise ϵ and the sample $x_{t+\delta }$. However, we can also train them as denoising models and get back the original flow target by interpolating the model output with $z_{t+\delta }$. Last, the steps s can be sampled nonuniformly, e.g., by using the sigmoid of a normal distribution as done in Stable Diffusion 3 (*21*), which we also use in our method.

#### Sampling diffusion-based models

For both DDPMs and flow matching models, samples can be obtained by starting with a variable z∼N(0,I) and iteratively moving it toward the data distribution by using the denoising model as target, giving rise to a neural ODE (ordinary differential equation) (*64*, *65*). For flow matching models, the ODE is $dz_{s}=\left[g_{\theta }\left(z_{s}\right)-z_{s}\right]\mathrm{ds}$, where $g_{\theta }$ is the trained denoiser. The simplest and most common choice to solve that ODE is an Euler scheme with the IC z(0)∼N(0,I) and a constant step size, which is the one we use in this paper. The number of discretization steps used in that process can be freely chosen, which we set to 25 based on initial experiments.

#### Learning diffusion–based models on spatiotemporal data

Since the underlying physical model used to produce the ERA5 dataset is deterministic, aleatoric uncertainty is driven mainly by the data assimilation process and the equirectangular sampling at 1.5° instead of the higher-resolution natural representation of the dataset. At a lead time of 24 hours, the uncertainty is rather small, as demonstrated by the very good skill that deterministic ML models have on this timescale. As a result, the conditional distribution $p\left(x_{t+\delta }|x_{t}\right)$ is narrowly centered around its expectation $E\left[x_{t+\delta }|x_{t}\right]$. Therefore, the states can be decomposed as

$$x_{t+\delta }=E\left[x_{t+\delta }|x_{t}\right]+r_{t+\delta }$$ (4)

where $r_{t+\delta }$ is a centered residual [$E\left(r_{t+\delta }|x_{t}\right)=0$] with a relatively small variance compared to the unconditional variance of states ${\left(x_{t}\right)}_{t\in D}$.

Training a deterministic model $f_{\theta }$ with MSE loss approximates the expectation term $E\left[x_{t+\delta }|x_{t}\right]$, because it is the optimal minimizer of the MSE objective

$$E\left[x_{t+\delta }|x_{t}\right]={\text{argmin}}_{M}\;E_{x_{t+\delta }∼p\left(x_{t+\delta }|x_{t}\right)}∥M-x_{t+\delta }{∥}^{2}$$ (5)

This means that we can use any deterministic model $f_{\theta }$ trained with MSE loss to approximate the expectation term or even an ensemble average of such models. Then, given a choice of $f_{\theta }$, we train a flow matching model $g_{\theta }$ to model residual data ${\tilde{r}}_{t+\delta }=\left[x_{t+\delta }-f_{\theta }\left(x_{t}\right)\right]/\sigma$ renormalized to unit variance, using the denoising loss function

$$L=E_{x_{t}\in D,s∼U\left[0,1\right],\epsilon ∼N\left(0,I\right)}\;\gamma_{s}∥g_{\theta }\left(x_{t},\left(1-s\right){\tilde{r}}_{t+\delta }+\mathrm{s\epsilon},f_{\theta }\left(x_{t}\right)\right)-{\tilde{r}}_{t+\delta }{∥}_{S}^{2}$$ (6)

Last, ensemble predictions are generated by sampling the generative model and adding it to the deterministic prediction $f_{\theta }\left(x_{t}\right)$. Forecasts at longer lead times are obtained by repeating this process autoregressively. Even if the 24-hour stochastic residual has a small magnitude, it is critical to model this residual as well as possible to correctly capture the dynamics at longer lead times.

The advantages of this decomposition are threefold: (i) reduce the computational complexity of training weather generative models by factorizing the common work between trainings, (ii) leverage state-of-the-art deterministic models trained with a very high computational budget, and cheaply adapt them to a generative setting; (iii) the residual data have a simpler structure that can translate to a diffusion sampling ODE with straighter paths, which generally results in better sample quality with fewer sampling steps. In the remainder of the paper, we call residual data or residuals the data where the output of a deterministic model has been removed and refer to this deterministic model as the underlying deterministic model. We will call residual generative model the model that is trained on residuals, as opposed to the generative model that makes complete forecasts by summing outputs from the deterministic and residual generative model.

#### Overcoming difficulties arising from residual modeling

In our experiments, we have observed overfitting of our deterministic models, with a validation and test error a few percent higher than the training error (visible in fig. S1 in section S4.1 of the Supplementary Materials). As a result, the distribution of residuals is different on the test set compared to the training set. A manifestation of this is that residuals have a higher norm on the test set compared to the training set, because the norm of residuals corresponds to the error of the deterministic model used to compute them. Therefore, the generative model has to produce residuals at test time from a slightly different distribution compared to what it was trained on.

The main problem that arises from this overfitting issue is underdispersion. Our residual generative model learns to model centered data on the training set, which means that the ensemble mean of residuals will be 0. Therefore, the ensemble mean matches the prediction of the underlying deterministic model. Consequently, the ensemble mean RMSE corresponds to the error of the underlying deterministic model, which is higher on the test set than on the training set. The ensemble mean RMSE being the discriminator of the spread-skill ratio (see section S3), this results in spread-skill ratios below one, hence underdispersion.

To mitigate this issue, we introduce OOD fine-tuning (for OOD), where we fine-tune the generative model on data that were not used for training the deterministic model. Residuals coming from these data source have a distribution closer to that which should be modeled on the test set. We also introduce noise scaling, which consists of scaling the initial noise for the sampling process, with a coefficient slightly higher than 1. The generative model then projects this noise distribution of a slightly higher variance, to an output distribution that also has a slightly higher variance, better matching the expected dispersion of members. In the experiments, we use a noise scaling coefficient of 1.05, which roughly corresponds to the percentage of overfitting observed for our underlying deterministic models.

#### Protocol for training ArchesWeatherGen

To sum up our training protocol for ArchesWeatherGen, we first choose a base deterministic model $f_{\theta }$, which approximates the conditional mean $E\left[x_{t+\delta }|x_{t}\right]$. By default, we use an ensemble of four ArchesWeather-M models, which better approximates this expectation compared to individual models by removing some biases due to neural network initialization. Then, we train a flow matching model on the renormalized residual values $r_{t+\delta }=\left[x_{t+\delta }-f_{\theta }\left(x_{t}\right)\right]/\sigma$, in two phases:

1) Phase 1: Train the denoising model on 1979–2018 data for 200,000 gradient update steps.

2) Phase 2 (OOD fine-tuning): Fine-tune on 2019 data for 60,000 steps, to adapt the generative model to residuals of higher norm corresponding to data not overfitted by the underlying deterministic model.

Because this flow matching model is used in a neural ODE at inference time, generating a single sample requires many calls to the neural network (25 in our case). That is why we chose the ArchesWeather-S architecture to further reduce inference costs.

### Evaluation

We evaluate deterministic models with the latitude-weighted RMSE. For generative models, we use CRPS, ensemble mean RMSE, energy score, Brier score (with a 1% threshold), and spread-skill ratio. Because of a statistical bias in the estimation of these scores due to a finite number of ensemble members, we use the fair version of these metrics that corresponds to their theoretical value in the limit of an infinite ensemble. This allows us to compare scores for ensembling methods with different ensemble sizes. We indicate the fair version of these metrics by prefixing them with the letter f, e.g., fCRPS for the fair CRPS (*66*). Below, we give a brief introduction to each of the metric that we use to evaluate weather models. The exact formula for each metric is presented in section S3 of the Supplementary Materials.

The ensemble mean RMSE is the RMSE between the ensemble mean and the ground truth atmospheric state (from ERA5) after a given lead time. While there is no ground truth for what the ensemble mean should be since there is a single ground truth state in ERA5, this metric still captures how well the ensemble mean match the expectation of the distribution: It decreases as the distance between the ensemble mean and the true distribution mean decreases. Unlike in single-member prediction, there is no trade-off between this metric and smoothness: An ensemble mean can display the correct smoothness (reflecting uncertainty) with members that are all physically consistent.

The continuous ranked probability score (*67*) is a widely used metric for probabilistic forecasts, which allows us to assess whether the pointwise marginal distributions predicted by a model correctly match the ground truth marginal distributions. Given that we are interested in the conditional probability distribution given the current state $x_{t}$, there is only one sample of the transition distribution (the following state $x_{t+\delta }$ in ERA5), but we can still compute the CRPS with this unique sample and average over all ICs in the test set. For the same lead time as training (24 hours in our case), the CRPS only evaluates whether the marginal distributions are correct and could be optimized without producing a physically consistent prediction. However, for ML models that are used in an autoregressive manner, reproducing the correct marginals at longer lead times requires producing physically consistent predictions at intermediate lead times; otherwise, the models are run on OOD inputs which degrade the trajectories. Hence, CRPS it is a representative metric of the quality of model outputs, beyond marginal distributions.

Brier scores (*68*) are a measure of the forecasting skill for extreme events. Given a quantile threshold q, they measure whether the empirical probability of a physical variable exceeding a threshold q is close to the ground truth (1 if the threshold was exceeded in ERA5, 0 otherwise). Here, we compute Brier scores using thresholds of <1 and >99% quantiles of the 1990–2018 climatology and then averaged together to get a unique score for the 1% tails of the distribution.

Spread-skill ratio is a measure of the dispersion of an ensemble, which assesses whether the predicted SD across members matches the ensemble mean RMSE, which should be the case for a perfect prediction (*69*). The model is underdispersive with a spread-skill ratio of less than 1 and overdispersive with a spread-skill ratio greater than 1, assuming relatively small forecast bias (*70*).

#### Summary metrics

For all metrics, latitude reweighting is used to account for the sampling distortion introduced by the equirectangular projection, so that the contribution of each pixel is proportional to its area on the sphere. We focus on evaluating key headline variables: *Z500*, *Q700*, *T850*, *U850*, *V850*, *T2m*, *SP*, *U10m*, and *V10m*. We also use skill scores to compare metrics to a reference model, allowing us to assess relative performance with respect to this model. For skill scores, higher is better: An RMSE skill score of 5% means that the evaluated model gets an RMSE score 5% lower compared to the reference model. Skill scores have the additional benefit to be dimensionless and therefore can be averaged, a property that we use to get a representative score across all variables. For instance with RMSE, we define the global RMSE skill score as the average

$$\text{RMSEss}\left(\text{model}\right)=\frac{1}{∥V∥}\sum_{v}\left[1-\frac{{\text{RMSE}}_{v}\left(\text{model}\right)}{{\text{RMSE}}_{v}\left(\text{ref}\right)}\right]$$ (7)

where ${\text{RMSE}}_{v}\left(X\right)$ is the RMSE of model X on physical variable v and ref is the reference model. We use the IFS models as reference, with HRES for evaluating deterministic models and ENS for evaluating probabilistic models. In all cases, these reference models are evaluated using their own analysis data as ground truth instead of ERA5, following WeatherBench (*50*).

## Supplementary Materials

### This PDF file includes:

Sections S1 to S5 Figs. S1 to S9 References

## References

[1] H. Hersbach, B. Bell, P. Berrisford, S. Hirahara, A. Horányi, J. Muñoz-Sabater, J. Nicolas, C. Peubey, R. Radu, D. Schepers, A. Simmons, C. Soci, S. Abdalla, X. Abellan, G. Balsamo, P. Bechtold, G. Biavati, J. Bidlot, M. Bonavita, G. De Chiara, P. Dahlgren, D. Dee, M. Diamantakis, R. Dragani, J. Flemming, R. Forbes, M. Fuentes, A. Geer, L. Haimberger, S. Healy, R. J. Hogan, E. Hólm, M. Janisková, S. Keeley, P. Laloyaux, P. Lopez, C. Lupu, G. Radnoti, P. de Rosnay, I. Rozum, F. Vamborg, S. Villaume, J.-N. Thépaut, The ERA5 global reanalysis. Q. J. Roy. Meteorol. Soc. 146, 1999–2049 (2020).

[2] K. Bi, L. Xie, H. Zhang, X. Chen, X. Gu, Q. Tian, Accurate medium-range global weather forecasting with 3D neural networks. Nature 619, 533–538 (2023). 37407823 10.1038/s41586-023-06185-3 PMC10356604

[3] J. Pathak, S. Subramanian, P. Harrington, S. Raja, A. Chattopadhyay, M. Mardani, T. Kurth, D. Hall, Z. Li, K. Azizzadenesheli, P. Hassanzadeh, K. Kashinath, A. Anandkumar, Fourcastnet: A global data-driven high-resolution weather model using adaptive fourier neural operators. arXiv:2202.11214 [physics.ao-ph] (2022).

[4] R. Lam, A. Sanchez-Gonzalez, M. Willson, P. Wirnsberger, M. Fortunato, F. Alet, S. Ravuri, T. Ewalds, Z. Eaton-Rosen, W. Hu, A. Merose, S. Hoyer, G. Holland, O. Vinyals, J. Stott, A. Pritzel, S. Mohamed, P. Battaglia, Learning skillful medium-range global weather forecasting. Science 382, 1416–1421 (2023). 37962497 10.1126/science.adi2336

[5] L. Chen, X. Zhong, F. Zhang, Y. Cheng, Y. Xu, Y. Qi, H. Li, FuXi: A cascade machine learning forecasting system for 15-day global weather forecast. Science 6, 190 (2023).

[6] T. Nguyen, R. Shah, H. Bansal, T. Arcomano, R. Maulik, R. Kotamarthi, I. Foster, S. Madireddy, A. Grover, Scaling transformer neural networks for skillful and reliable medium-range weather forecasting. Adv. Neural Inf. Process. Syst. 37, 68740–68771 (2024).

[7] E. Guo, M. Ahmed, Y. Sun, R. Mahendru, R. Yang, H. Cook, T. Leeuwenburg, B. Evans, FourCastNeXt: Improving fourcastnet training with limited compute. arXiv:2401.05584 [cs.CV] (2024).

[8] D. Kochkov, J. Yuval, I. Langmore, P. Norgaard, J. Smith, G. Mooers, M. Klöwer, J. Lottes, S. Rasp, P. Düben, S. Hatfield, P. Battaglia, A. Sanchez-Gonzalez, M. Willson, M. P. Brenner, S. Hoyer, Neural general circulation models for weather and climate. Nature 632, 1060–1066 (2024). 39039241 10.1038/s41586-024-07744-y PMC11357988

[9] C. Lessig, I. Luise, B. Gong, M. Langguth, S. Stadler, M. Schultz, AtmoRep: A stochastic model of atmosphere dynamics using large scale representation learning. arXiv:2308.13280 [physics.ao-ph] (2023).

[10] A. Dosovitskiy, L. Beyer, A. Kolesnikov, D. Weissenborn, X. Zhai, T. Unterthiner, M. Dehghani, M. Minderer, G. Heigold, S. Gelly, J. Uszkoreit, N. Houlsby, An image is worth 16x16 words: Transformers for image recognition at scale (2021), https://openreview.net/forum?id=YicbFdNTTy.

[11] M. Bonavita, On some limitations of current machine learning weather prediction models. Geophys. Res. Lett. 51, e2023GL107377 (2024).

[12] A. Ramesh, M. Pavlov, G. Goh, S. Gray, C. Voss, A. Radford, M. Chen, I. Sutskever, “Zero-shot text-to-image generation,” in *International Conference on Machine Learning* (PMLR, 2021), pp. 8821–8831.

[13] P. Esser, R. Rombach, B. Ommer, “Taming transformers for high-resolution image synthesis,” in *Proceedings of the IEEE/CVF Conference on Computer Vision and Pattern Recognition* (IEEE, 2021), pp. 12,873–12,883.

[14] C. Saharia, W. Chan, S. Saxena, L. Li, J. Whang, E. Denton, S. K. S. Ghasemipour, B. K. Ayan, S. S. Mahdavi, R. G. Lopes, T. Salimans, J. Ho, D. J. Fleet, M. Norouzi, Photorealistic text-to-image diffusion models with deep language understanding. Adv. Neural Inf. Process. Syst. 35, 36479–36494 (2022).

[15] R. Rombach, A. Blattmann, D. Lorenz, P. Esser, B. Ommer, “High-resolution image synthesis with latent diffusion models,” in *Proceedings of the IEEE/CVF Conference on Computer Vision and Pattern Recognition* (IEEE, 2022), pp. 10,684–10,695.

[16] I. Price, A. Sanchez-Gonzalez, F. Alet, T. R. Andersson, A. El-Kadi, D. Masters, T. Ewalds, J. Stott, S. Mohamed, P. Battaglia, R. Lam, M. Willson, Probabilistic weather forecasting with machine learning. Nature 637, 84–90 (2025). 39633054 10.1038/s41586-024-08252-9 PMC11666454

[17] S. Shang, Z. Shan, G. Liu, L. Wang, X. Wang, Z. Zhang, J. Zhang, “Resdiff: Combining cnn and diffusion model for image super-resolution,” in *Proceedings of the AAAI Conference on Artificial Intelligence* (AAAI Press, 2024), vol. 38, pp. 8975–8983.

[18] M. Mardani, N. Brenowitz, Y. Cohen, J. Pathak, C.-Y. Chen, C.-C. Liu, A. Vahdat, M. A. Nabian, T. Ge, A. Subramaniam, K. Kashinath, J. Kautz, M. Pritchard, Residual corrective diffusion modeling for km-scale atmospheric downscaling. Commun. Earth Environ. 6, 124 (2025).

[19] J. Ho, A. Jain, P. Abbeel, Denoising diffusion probabilistic models. Adv. Neural. Inf. Process. Syst. 33, 6840–6851 (2020).

[20] T. Karras, M. Aittala, T. Aila, S. Laine, Elucidating the design space of diffusion-based generative models. Adv. Neural. Inf. Process. Syst. 35, 26565–26577 (2022).

[21] P. Esser, S. Kulal, A. Blattmann, R. Entezari, J. Müller, H. Saini, Y. Levi, D. Lorenz, A. Sauer, F. Boesel, D. Podell, T. Dockhorn, Z. English, K. Lacey, A. Goodwin, Y. Marek, R. Rombach, “Scaling rectified flow transformers for high-resolution image synthesis,” in *ICML’24: Proceedings of the 41st International Conference on Machine Learning* (2024), pp. 12,606–12,633.

[22] Y. Lipman, R. T. Chen, H. Ben-Hamu, M. Nickel, M. Le, “Flow matching for generative modeling,” in *11th International Conference on Learning Representations* (2023), pp. 28.

[23] J. Song, C. Meng, S. Ermon, “Denoising diffusion implicit models,” *International Conference on Learning Representations* (2020).

[24] A. Vaswani, N. Shazeer, N. Parmar, J. Uszkoreit, L. Jones, A. N. Gomez, L. Kaiser, I. Polosukhin, Attention is all you need. Advances in Neural Information Processing Systems (2017), vol 30, pp. 5998–6008.

[25] Z. Liu, H. Hu, Y. Lin, Z. Yao, Z. Xie, Y. Wei, J. Ning, Y. Cao, Z. Zhang, L. Dong, F. Wei, B. Guo, “Swin transformer v2: Scaling up capacity and resolution,” in *Proceedings of the IEEE/CVF Conference on Computer Vision and Pattern Recognition* (IEEE, 2022), pp. 12,009–12,019.

[26] L. Chen, F. Du, Y. Hu, Z. Wang, F. Wang, “Swinrdm: Integrate swinrnn with diffusion model towards high-resolution and high-quality weather forecasting,” in *Proceedings of the AAAI Conference on Artificial Intelligence* (AAAI Press, 2023), vol. 37, pp. 322–330.

[27] X. Zhong, L. Chen, J. Liu, C. Lin, Y. Qi, H. Li, FuXi-Extreme: Improving extreme rainfall and wind forecasts with diffusion model. Sci. China Earth Sci. 67, 3696–3708 (2024).

[28] L. Li, R. Carver, I. Lopez-Gomez, F. Sha, J. Anderson, Generative emulation of weather forecast ensembles with diffusion models. Sci. Adv. 10, eadk4489 (2024). 38552014 10.1126/sciadv.adk4489 PMC10980268

[29] C. Brochet, G. Moldovan, L. Raynaud, M. Plu, “Using state-of-the-art generative neural networks for high-resolution NWP ensemble emulation” (Tech. Rep., Copernicus Meetings, 2023).

[30] P. Srivastava, R. Yang, G. Kerrigan, G. Dresdner, J. McGibbon, C. Bretherton, S. Mandt, Probabilistic precipitation downscaling with optical flow-guided diffusion. arXiv:2312.06071v1 [cs.CV] (2023).

[31] R. A. Watt, L. A. Mansfield, Generative diffusion-based downscaling for climate. arXiv:2404.17752 [physics.ao-ph] (2024).

[32] E. Tomasi, G. Franch, M. Cristoforetti, Can AI be enabled to perform dynamical downscaling? A latent diffusion model to mimic kilometer-scale COSMO5.0_CLM9 simulations. EGUsphere 18, 2051–2078 (2024).

[33] Q. Han, X. Jiang, Y. Zhao, X. Wang, Z. Li, R. Zhang, Diffusion-model-based downscaling of observed sea surface height over the kuroshio extension since 2000. Atmos. 16, 570 (2025).

[34] I. Lopez-Gomez, Z. Y. Wan, L. Zepeda-Núñez, T. Schneider, J. Anderson, F. Sha, Dynamical-generative downscaling of climate model ensembles. Proc. Natl. Acad. Sci. U.S.A. 122, e2420288122 (2025). 40279391 10.1073/pnas.2420288122 PMC12054837

[35] D. Yu, X. Li, Y. Ye, B. Zhang, C. Luo, K. Dai, R. Wang, X. Chen, “Diffcast: A unified framework via residual diffusion for precipitation nowcasting,” in *Proceedings of the IEEE/CVF Conference on Computer Vision and Pattern Recognition* (IEEE, 2024), pp. 27758–27767.

[36] Z. Zhao, X. Dong, Y. Wang, C. Hu, Advancing realistic precipitation nowcasting with a spatiotemporal transformer-based denoising diffusion model. IEEE Trans. Geosci. Remote Sens. 62, 1–15 (2024).

[37] J. Gong, L. Bai, P. Ye, W. Xu, N. Liu, J. Dai, X. Yang, W. Ouyang, “CasCast: Skillful high-resolution precipitation nowcasting via cascaded modelling,” in *International Conference on Machine Learning* (PMLR, 2024).

[38] H. Addison, E. Kendon, S. Ravuri, L. Aitchison, P. A. Watson, Machine learning emulation of precipitation from km-scale regional climate simulations using a diffusion model. arXiv:2407.14158 [physics.ao-ph] (2024).

[39] C. Guilloteau, G. Kerrigan, K. Nelson, G. Migliorini, P. Smyth, R. Li, E. Foufoula-Georgiou, A generative diffusion model for probabilistic ensembles of precipitation maps conditioned on multisensor satellite observations. IEEE Trans. Geosci. Remote Sens. 63, 1–15 (2025).

[40] P. Nath, P. Shukla, S. Wang, C. Quilodrán-Casas, Forecasting tropical cyclones with cascaded diffusion models. arXiv:2310.01690 [physics.ao-ph] (2023).

[41] C. Huang, P. Mu, C. Bai, P. A. Watson, “TCP-diffusion: A multi-modal diffusion model for global tropical cyclone precipitation forecasting with change awareness,” *International Conference on Machine Learning* (PMLR, 2024); https://openreview.net/forum?id=9cOtYlD5UA.

[42] T. S. Finn, C. Durand, A. Farchi, M. Bocquet, J. Brajard, Towards diffusion models for large-scale sea-ice modelling. arXiv:2406.18417 [cs.LG] (2024).

[43] L. Huang, L. Gianinazzi, Y. Yu, P. D. Dueben, T. Hoefler, “DiffDA: A diffusion model for weather-scale data assimilation,” *International Conference on Machine Learning* (PMLR, 2024); https://openreview.net/forum?id=vhMq3eAB34.

[44] F. Rozet, G. Louppe, Score-based data assimilation. Adv. Neural Inf. Process Syst. 36, 40521–40541 (2023).

[45] P. Manshausen, Y. Cohen, J. Pathak, M. Pritchard, P. Garg, M. Mardani, K. Kashinath, S. Byrne, N. Brenowitz, Generative data assimilation of sparse weather station observations at kilometer scales. arXiv:2406.16947 [cs.LG] (2024).

[46] C. Esteves, J.-J. Slotine, A. Makadia, Scaling spherical CNNs. arXiv:2306.05420 [cs.LG] (2023).

[47] J. A. Weyn, D. R. Durran, R. Caruana, Improving data-driven global weather prediction using deep convolutional neural networks on a cubed sphere. J. Adv. Model. Earth Syst. 12, e2020MS002109 (2020).

[48] K. Pandey, J. Pathak, Y. Xu, S. Mandt, M. Pritchard, A. Vahdat, M. Mardani, “Heavy-tailed diffusion models,” in *International Conference on Learning Representations* (2024); https://openreview.net/forum?id=tozlOEN4qp.

[49] L. Isaksen, M. Bonavita, R. Buizza, M. Fisher, J. Haseler, M. Leutbecher, L. Raynaud, *Ensemble of Data Assimilations at ECMWF* (ECMWF, 2010).

[50] S. Rasp, S. Hoyer, A. Merose, I. Langmore, P. Battaglia, T. Russell, A. Sanchez-Gonzalez, V. Yang, R. Carver, S. Agrawal, M. Chantry, Z. Ben Bouallegue, P. Dueben, C. Bromberg, J. Sisk, L. Barrington, A. Bell, F. Sha, WeatherBench 2: A benchmark for the next generation of data-driven global weather models. J. Adv. Model. Earth Syst. 16, e2023MS004019 (2024).

[51] Z. Liu, Y. Lin, Y. Cao, H. Hu, Y. Wei, Z. Zhang, S. Lin, B. Guo, “Swin transformer: Hierarchical vision transformer using shifted windows,” in *Proceedings of the IEEE/CVF International Conference on Computer Vision* (IEEE, 2021), pp. 10,012–10,022.

[52] J. Ho, N. Kalchbrenner, D. Weissenborn, T. Salimans, Axial attention in multidimensional transformers. arXiv:1912.12180 [cs.CV] (2019).

[53] M. Andrychowicz, L. Espeholt, D. Li, S. Merchant, A. Merose, F. Zyda, S. Agrawal, N. Kalchbrenner, Deep learning for day forecasts from sparse observations. arXiv:2306.06079 [physics.ao-ph] (2023).

[54] A. Aitken, C. Ledig, L. Theis, J. Caballero, Z. Wang, W. Shi, Checkerboard artifact free sub-pixel convolution: A note on sub-pixel convolution, resize convolution and convolution resize. arXiv:1707.02937 [cs.CV] (2017).

[55] N. Shazeer, Glu variants improve transformer. arXiv:2002.05202 [cs.LG] (2020).

[56] H. Touvron, T. Lavril, G. Izacard, X. Martinet, M.-A. Lachaux, T. Lacroix, B. Rozière, N. Goyal, E. Hambro, F. Azhar, A. Rodriguez, A. Joulin, E. Grave, G. Lample, Llama: Open and efficient foundation language models. arXiv:2302.13971 [cs.CL] (2023).

[57] E. Perez, F. Strub, H. De Vries, V. Dumoulin, A. Courville, “Film: Visual reasoning with a general conditioning layer,” in *Proceedings of the AAAI Conference on Artificial Intelligence* (AAAI Press, 2018), vol. 32.

[58] A. Krogh, J. Vedelsby, Neural network ensembles, cross validation, and active learning. Adv. Neural Inf. Process. Syst. 7, 231–238 (1994).

[59] Z. Ben Bouallegue, Accuracy versus activity. ECMWF website (2024), 10.21957/8b50609a0f; https://www.ecmwf.int/en/about/media-centre/aifs-blog/2024/accuracy-versus-activity.

[60] J. Sohl-Dickstein, E. Weiss, N. Maheswaranathan, S. Ganguli, “Deep unsupervised learning using nonequilibrium thermodynamics,” in *International Conference on Machine Learning (*PMLR, 2015), pp. 2256–2265.

[61] A. Q. Nichol, P. Dhariwal, “Improved denoising diffusion probabilistic models,” in International Conference on Machine Learning (PMLR, 2021), pp. 8162–8171.

[62] L. Weng, What are diffusion models? (2021), p. 21, https://lilianweng.github.io/.

[63] H. Yu, L. Shen, J. Huang, H. Li, F. Zhao, Unmasking Bias in Diffusion Model Training. arXiv preprint arXiv:2310.08442 (2023).

[64] P. Dhariwal, A. Nichol, Diffusion models beat gans on image synthesis. Adv. Neural Inf. Process. Syst. 34, 8780–8794 (2021).

[65] G. Couairon, J. Verbeek, H. Schwenk, M. Cord, “DiffEdit: Diffusion-based semantic image editing with mask guidance,” in *International Conference on Learning Representations* (2023), https://openreview.net/forum?id=3lge0p5o-M-.

[66] C. Ferro, Fair scores for ensemble forecasts. Q. J. Roy. Meteorol. Soc. 140, 1917–1923 (2014).

[67] T. Gneiting, A. E. Raftery, Strictly proper scoring rules, prediction, and estimation. J. Am. Stat. Assoc. 102, 359–378 (2007).

[68] M. S. Roulston, Performance targets and the Brier score. Met. Apps. 14, 185–194 (2007).

[69] V. Fortin, M. Abaza, F. Anctil, R. Turcotte, Why should ensemble spread match the RMSE of the ensemble mean? J. Hydrometeorol. 15, 1708–1713 (2014).

[70] T. M. Hamill, Interpretation of rank histograms for verifying ensemble forecasts. Mon. Wea. Rev. 129, 550–560 (2001).

[71] H. Hersbach, B. Bell, P. Berrisford, G. Biavati, A. Horányi, J. Muñoz Sabater, J. Nicolas, C. Peubey, R. Radu, I. Rozum, D. Schepers, A. Simmons, C. Soci, D. Dee, J.-N. Thépaut, ERA5 hourly data on single levels from 1940 to present. *Copernicus Climate Change Service (C3S) Climate Data Store (CDS)* (2023), accessed on 19 December 2024; 10.24381/cds.adbb2d47.

[72] G. Deepmind, WeatherNext, a family of AI models from Google DeepMind and Google Research, produces state-of-the-art weather forecasts. (2025), https://deepmind.google/science/weathernext/#access-weathernext.

[73] G. Couairon, R. Singh, GeoArches (2024); https://github.com/INRIA/geoarches.

[74] D. P. Kingma, J. Ba, Adam: A method for stochastic optimization. arXiv:1412.6980 [cs.LG] (2014).

[75] L. AI, A100 vs V100 Deep Learning Benchmarks (2021); https://lambda.ai/blog/nvidia-a100-vs-v100-benchmarks.

[76] N. P. Jouppi, G. Kurian, S. Li, P. Ma, R. Nagarajan, L. Nai, N. Patil, S. Subramanian, A. Swing, B. Towles, C. Young, X. Zhou, Z. Zhou, D. Patterson, “Tpu v4: An optically reconfigurable supercomputer for machine learning with hardware support for embeddings,” in *Proceedings of the 50th Annual International Symposium on Computer Architecture* (IEEE / ACM, 2023), pp. 1–14.

[77] G. C. Blog, Enabling next-generation AI workloads: Announcing TPU v5p and AI Hypercomputer (2023), https://cloud.google.com/blog/products/ai-machine-learning/introducing-cloud-tpu-v5p-and-ai-hypercomputer.

[78] C. A. T. Ferro, Comparing probabilistic forecasting systems with the brier score. Weather Forecast. 22, 1076–1088 (2007).

[79] P. Bauer, A. Thorpe, G. Brunet, The quiet revolution of numerical weather prediction. Nature 525, 47–55 (2015). 26333465 10.1038/nature14956

[80] O. Talagrand, “Evaluation of probabilistic prediction systems,” in *Workshop Proceedings “Workshop on Predictability,” 20–22 October 1997*, (ECMWF, Reading, UK 1999).

