# Supplementary material for: ArchesWeatherGen: Skillful and compute-efficient probabilistic weather forecasting with machine learning
Source: Sci Adv. 2026 Apr 22;12(17):eadx2372. doi: 10.1126/sciadv.adx2372 (PMC13101865; doi:10.1126/sciadv.adx2372)
Supplement: Supplementary file 1 — Sections S1 to S5 Figs. S1 to S9 References [file sciadv.adx2372_sm.pdf]

Science Advances
AAAS

Supplementary Materials for

ArchesWeatherGen: Skillful and compute-efficient probabilistic weather forecasting with machine learning

Guillaume Couairon *et al.*

Corresponding author: Guillaume Couairon, guillaume.couairon@gmail.com

Sci. Adv. **12**, eadx2372 (2026)
DOI: 10.1126/sciadv.adx2372

This PDF file includes:

Sections S1 to S5
Figs. S1 to S9
References

1 Training details

We denote $(\mathbf{x}_t)_{t\in\mathcal{D}}$ the historical trajectory of ERA5, indexed by time t. Input states $\mathbf{x}_t$ are normalized to zero mean and unit variance on a per-variable and per-level basis, using statistics of the training set 1979-2018.

Following GraphCast, we scale the training loss with coefficients proportional to the air density, to give more importance to variables closer to the surface. We also use the same reweighting of the surface variables with a coefficient of 1 for 2m temperature, and 0.1 for wind components and mean surface pressure.

We train all our models with the AdamW optimizer (*74*). The batch size is 4 and the optimizer parameters are a learning rate of 3e-4, beta parameters ($\beta_1 = 0.9, \beta_2 = 0.98$) and a weight decay of 0.05. The learning rate is increased linearly for the first 5000 steps, then decayed with a cosine schedule for the remaining steps.

2 Comparison with state-of-the-art

Deterministic models For deterministic models (except Stormer), RMSE scores at 1.5º are taken from WeatherBench2 (*50*). For Stormer (*6*), we evaluate outputs provided by the authors at 1.4º resolution. Stormer is a ~300M parameters model trained to forecast ERA5 variables at multiple lead times simultaneously: 6h, 12h and 24h. To make a 24h lead time forecast, Stormer uses all possible combinations of lead times as conditioning: 24h, 12h-12h, 12h-6h-6h, 6h-12h-6h, 6h-6h-12h, 6h-6h-6h-6h, and averages all trajectories. This base model is run 16 times with different lead time conditioning to make a 24h forecast. Our ensemble model requires four model forwards with $\sim 336M$ total parameters. Please see the paper

(*6*) for more details on Stormer.

Probabilistic models For evaluating probabilistic models, when possible we used the 2020 forecasts accessible from WeatherBench, which was the case for IFS ENS and NeuralGCM. We then re-compute our metrics on these forecasts. For GenCast 0.25º and 1.0º, we used forecasts gracefully provided by the authors.

3 Evaluation

3.1 Computational cost metrics

To compare the computational cost of training on different GPUs, we convert all of them to the cost of training on V100 GPUs. Here is our methodology. For converting between the different GPUs (V100 vs A100 vs H100), we empirically found that our trainings were roughly twice as fast on an H100 compared to an A100, and also twice as fast on an A100 compared to a V100. While the relative speed of these models depends on the machine learning task (e.g. training convolutional neural networks or transformer-based language models), these figures are consistent with the benchmarks that we found online; For instance, in a benchmark by the Lambda Cloud company (*75*), the A100 was estimated to be 1.6x-2.2x faster than the V100 for training vision models.

We only had access to GPUs and not TPUs, so to estimate the computational cost of TPU-trained models (GraphCast and NeuralGCM), we based our estimates on the original article introducing the TPUv4 (*76*), which estimates that the TPUv4 is

1.2x-1.7x faster than an A100, which we rounded to a relative cost of 1 TPUv4/day = 1.5 A100/day = 3 V100/day. For TPUv5, we used an estimation from the Google Cloud blog (*77*) that the TPUv5p is roughly twice faster than the TPUv4, hence we converted into 1 TPUv5p/day = 2 TPUv4/day = 6 V100/day.

3.2 Skill Metrics

We recall that we consider weather states consisting of six upper air variables (temperature, geopotential, specific humidity, wind components U, V and W) sampled on a latitude-longitude grid at 13 pressure levels, and four surface variables (2m temperature, mean sea level pressure, 10m wind U and V). We note the historical trajectory of these weather states $\mathcal{D} = (\mathbf{x}_t)_{t \in \mathcal{T}}$ indexed by the time variable t. For a given initialization time t and a forecasting method that predicts an ensemble of M forecasts, we note $\hat{\mathbf{x}}^{v,i}_{t,\delta}$ the forecast of the i-th ensemble member at lead time δ, for the physical variable v. The ground truth target is $\mathbf{x}^{v}_{t+\delta}$.

The ensemble mean is an average of the members' predictions:

$$\bar{\mathbf{x}}^{v}_{t,\delta} = \frac{1}{M} \sum_{i=1}^{M} \hat{\mathbf{x}}^{v,i}_{t,\delta} \tag{S1}$$

where we use a value of $M = 50$ in the main article.

We note $\|\cdot\|_1$ and $\|\cdot\|_2$, respectively, the L1-norm and L2-norm of a scalar field defined on the sphere. Since we only compute this average on fields sampled with the equirectangular projection, we use latitude weighting to account for area distortion.

In the next paragraphs, we give the explicit formula for each of the statistical estimators that we use as metrics. Unless otherwise specified, we use the "fair"

or "debiased" version of these estimators, also explicitly defined in each of the following paragraphs. The fair estimators estimate the value of their biased counterparts in the limit of an infinite sample size, meaning that they can be compared across different sample sizes (although in the main article we only use $M = 50$ ensemble members). As a side note, we observed that when comparing skill scores with respect to a reference model, using biased or debiased gives extremely similar results, with a negligible difference.

Ensemble Mean RMSE The EnsembleMean RMSE is defined as

$$\text{EnsembleMeanRMSE}(v, \delta) := \sqrt{\frac{1}{T} \sum_t \|\bar{\mathbf{x}}^v_{t,\delta} - \mathbf{x}^v_{t+\delta}\|_2^2} \tag{S2}$$

In the main article, we use the debiased version of this estimator from WeatherBench:

$$\text{FairEnsMeanRMSE}(v, \delta) := \sqrt{\frac{1}{T} \sum_t \left(\|\bar{\mathbf{x}}^v_{t,\delta} - \mathbf{x}^v_{t+\delta}\|_2^2 - \frac{1}{M(M-1)} \sum_i \|\bar{\mathbf{x}}^v_{t,\delta} - \hat{\mathbf{x}}^{v,i}_{t,\delta}\|_2^2 \right)} \tag{S3}$$

where t usually covers the range of initialization times in the test dataset (all 00z/12z initialization times for 2020 in our case).

CRPS and Energy Score We only use the "fair" version of the CRPS (*66*):

$$\text{CRPS}(v, \delta) := \frac{1}{T} \sum_t \left(\frac{1}{M} \sum_i \|\hat{\mathbf{x}}^{v,i}_{t,\delta} - \mathbf{x}^v_{t+\delta}\|_1 - \frac{1}{2M(M-1)} \sum_{i,j} \|\hat{\mathbf{x}}^{v,i}_{t,\delta} - \hat{\mathbf{x}}^{v,j}_{t,\delta}\|_1 \right) \tag{S4}$$

The Energy Score (ES) is the counterpart of the CRPS, using the $\|\cdot\|_2$ norm instead of the $\|\cdot\|_1$ norm.

Activity The activity is the location-based variance of the forecasts where climatology has been removed: with $\mathbf{c}_t^v$ being the climatology of variable v at time t (day of year/hour), activity is defined as

$$\text{Activity} := \frac{1}{T}\sum_t \frac{1}{M}\sum_i \left(\|(\mathbf{x}_{t,\delta}^{v,i} - \mathbf{c}_{t+\delta}^v) - \overline{(\mathbf{x}_{t,\delta}^{v,i} - \mathbf{c}_{t+\delta}^v)}\|_2^2 \right) \tag{S5}$$

where the overline represents the (latitude-weighted) spatial average.

Spread-Skill Ratio The spread of an ensemble forecast is simply defined with the unbiased variance estimator

$$\text{Spread}(v, \delta) := \sqrt{\frac{1}{T}\sum_t \frac{1}{M-1}\sum_i \|\hat{\mathbf{x}}_{t,\delta}^{v,i} - \bar{\mathbf{x}}_{t,\delta}^v\|_2^2} \tag{S6}$$

and the unbiased spread-skill ratio is defined as

$$\text{SpreadSkillRatio}(v, \delta) := \sqrt{\frac{M+1}{M}} \frac{\text{Spread}(v, \delta)}{\text{EnsMeanRMSE}(v, \delta)} \tag{S7}$$

The spread-skill ratio should be equal to 1 in the assumption of a perfect ensemble forecast.

Brier Score Let c_q^v be the q-percentile of climatology for physical variable v, the empirical probability that variable v exceeds threshold q for initialization time t and lead time δ is

$$p_{t,\delta,q}^v = \frac{1}{M}\sum_i \mathbb{1}(\hat{\mathbf{x}}_{t,\delta}^{v,i} > c_q^v) \tag{S8}$$

The Brier Score is then defined as:

$$\text{BrierScore}(v, \delta, q) := \frac{1}{T}\sum_t \|p_{t,\delta,q}^v - \mathbb{1}(\mathbf{x}_{t+\delta}^v > c_q^v)\|_2^2 \tag{S9}$$

We use the fair version of this estimator as defined in (*66, 78*):

$$\text{fairBrierScore}(v, \delta, q) := \frac{1}{T} \sum_t \left(\| p^v_{t,\delta,q} - \mathbb{1}(\mathbf{x}^v_{t+\delta} > c^v_q) \|_2^2 - \frac{1}{M-1} p^v_{t,\delta,q} (1 - p^v_{t,\delta,q}) \right) \tag{S10}$$

The Brier score for the 1% tails that we use in the main article is the average of the brier scores for the 1% and 99% climatology percentiles.

Skill scores and Summary Metrics For all metrics, we define a skill score for variable v as the relative metric improvement compared to a reference model, usually the IFS HRES or ENS. For instance, the RMSE skill score is defined as

$$\text{RMSE-SkillScore}_{model}(v, \delta) := 1 - \frac{\text{RMSE}_{model}(v, \delta)}{\text{RMSE}_{ref}(v, \delta)} \tag{S11}$$

and has no unit. Therefore, we can average it over variables to get a representative average over all physical variables:

$$\text{RMSE-SkillScore}_{model}(\delta) := \frac{1}{\|\mathcal{V}\|} \sum_v \text{RMSE-SkillScore}_{model}(v, \delta) \tag{S12}$$

4 Additional evaluation of our model

4.1 Motivation for recent-past fine-tuning

Figure S1 shows the forecasting error of some key variables for a base ArchesWeather-S model, that is not finetuned on the recent past, computed separately for each year in the training dataset. This illustrates a distribution shift in ERA5, motivating a bigger emphasis on recent data, that has a distribution closer to the test data (2020).

Figure S2 shows a breakdown of the forecasting error per spatial location, for the geopotential at 500hPa (*Z500*), u and v components of wind at 850 hPa

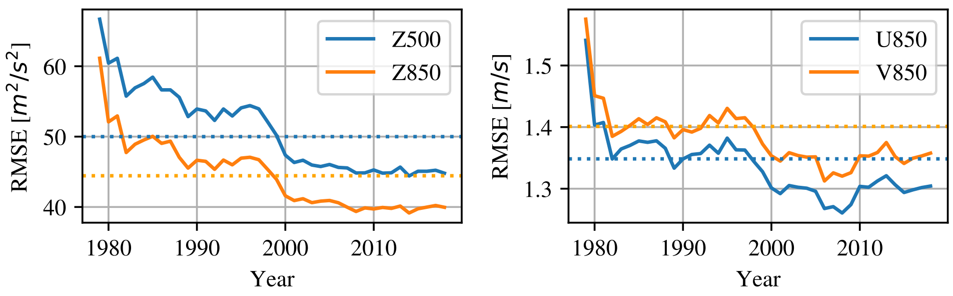

Figure S1: **Geopotential (left) and wind speed (right) RMSE of a model without multi-step fine-tuning, for each year in the training set**. The error is lower in the recent past, which we attribute to a more observed, constrained, and predictable dynamical system. Additionally, test RMSE (year 2020) are shown in dotted lines, which shows some overfitting compared to the scores in 2018 (last year in train set).

(*U850* and *V850*). We can see that there is a significant difference in forecasting errors between the Southern and Northern hemisphere before 2000 (especially on geopotential) and that this difference is much reduced after 2000. It is known that the increased amount of satellite Earth observations helped bridge the forecast error gap between the two hemispheres (*79*), and we observe the same trend here with a machine learning weather model trained on ERA5. Again, this illustrates a distribution shift after 2000 in ERA5, which motivates fine-tuning on the recent past. This phenomenon is again illustrated with the zonal mean forecasting error in Figure S3, computed separately for land points and ocean points. The discontinuity in the land errors corresponds to the Antarctic Circumpolar Current around 60ºS, where the error is the largest because of the strong winds in that area.

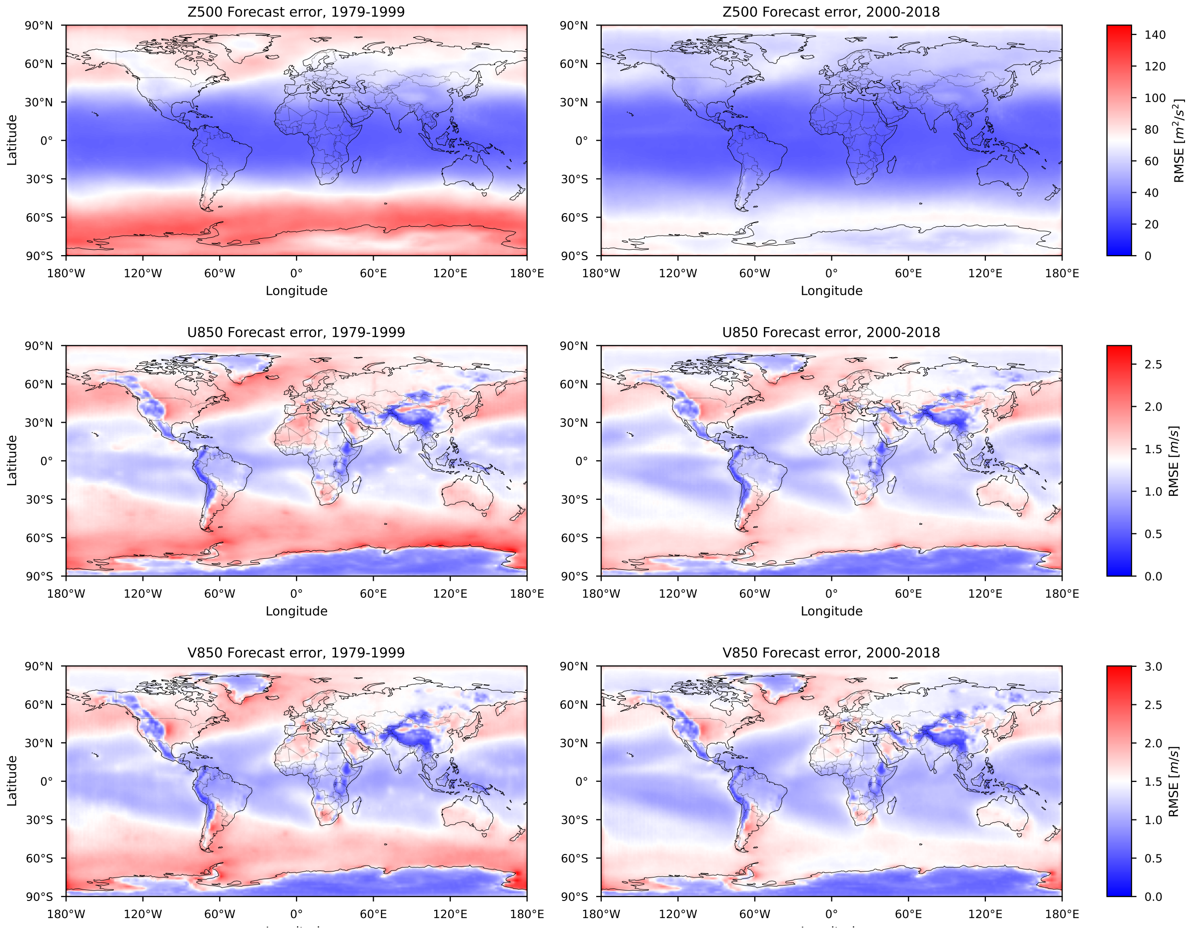

Figure S2: **Forecasting error of a non-finetuned ArchesWeather-S model, per spatial location averaged for the two periods 1979-1999 and 2000-2018**. We can see that the errors are smaller for the recent period, and that there is a significant skill difference between the Northern and Southern hemisphere before 2000, highlighting a distribution shift.

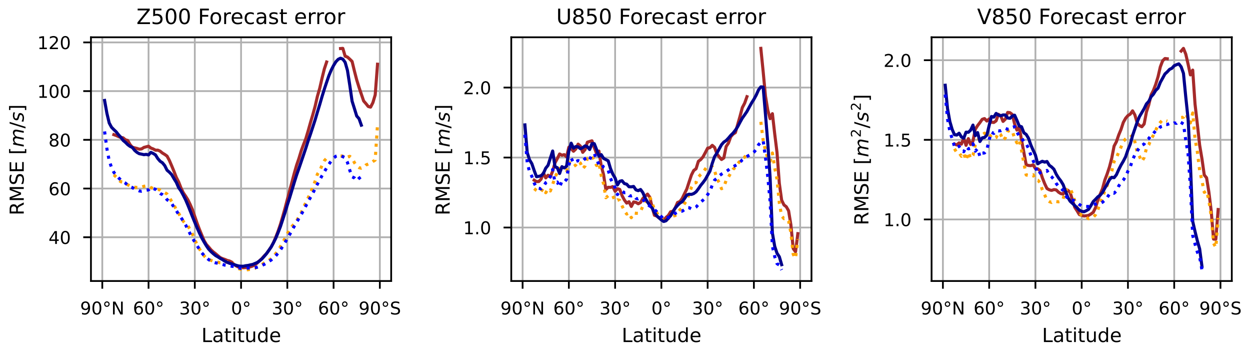

Figure S3: **Zonal mean forecasting error of a non-finetuned ArchesWeather-S model for the periods 1979-1999 (full line) and 2000-2018 (dashed line)**. The errors are computed separately for ocean points (blue) and land points (red). We can see that the errors are smaller for the recent period, in particular in the Southern hemisphere.

4.2 Activity of ArchesWeatherGen compared to other models

Activity is the standard deviation of the climatology-removed forecast across spatial location, which is a measure of how smooth (and hence, unphysical) the forecasts are.

Figure S4 shows that the activity of our ArchesWeatherGen is much closer to the ground truth compared to our deterministic models. We can see the activity of deterministic models decreasing as the lead time increases, which shows that smoothing is stronger at longer lead times. This effect is particularly strong for the ArchesWeather-Mx4 ensemble, because averaging the outputs of different models increases smoothing even more compared to a single RMSE-trained model.

Reliability of ensemble forecasts To validate the distributions of our generated ensembles, we use rank histograms (*80*), which consists of computing the rank of the ground truth among the M-members ensemble for each sample, and then calculating the histograms of these ranks, averaging over spatial locations. For

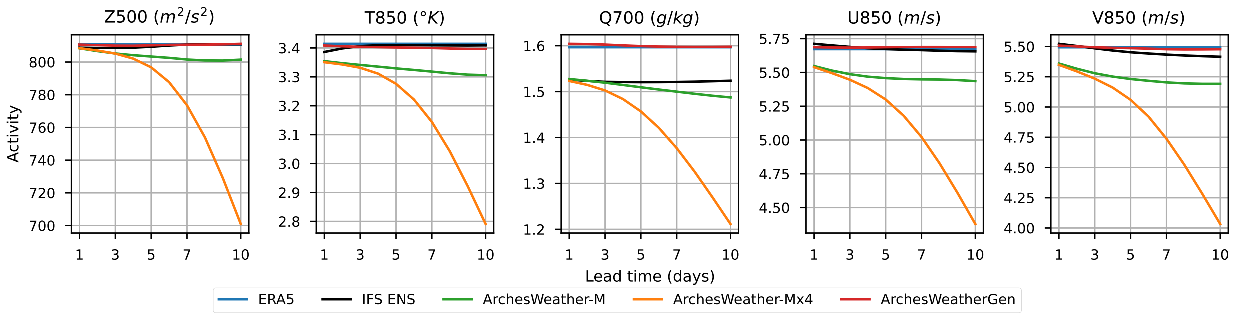

Figure S4: **Activity of our models for lead times up to 10 days.** ArchesWeatherGen's activity is very close to the ERA5 reference.

perfect ensemble forecasts, we expect flat rank histograms. Over-dispersive models are characterized by ∩-shaped rank histogram, and under-dispersive models by a ∪-shaped rank histogram. In Figure S5, we compare the rank histograms of ArchesWeatherGen, NeuralGCM and IFS ENS on upper-air variables. At 24h, ArchesWeather has almost perfectly flat histograms (very slightly under-dispersive for the extremes), much better than IFS ENS and NeuralGCM. At 7 days lead time, the rank histograms are not flat anymore, and slightly under-dispersive. Overall, they are flatter than IFS ENS, better than NeuralGCM for *Z500* an *Q700* and slightly worse on *T850, U850* and *V850* due to the remaining under-dispersiveness. We believe that this could be solved by adjusting the noise scaling parameter separately for each physical variable, which we did not investigate for simplicity of the approach.

Ablations of the generative model

In this section, we validate our design choices for ArchesWeatherGen.

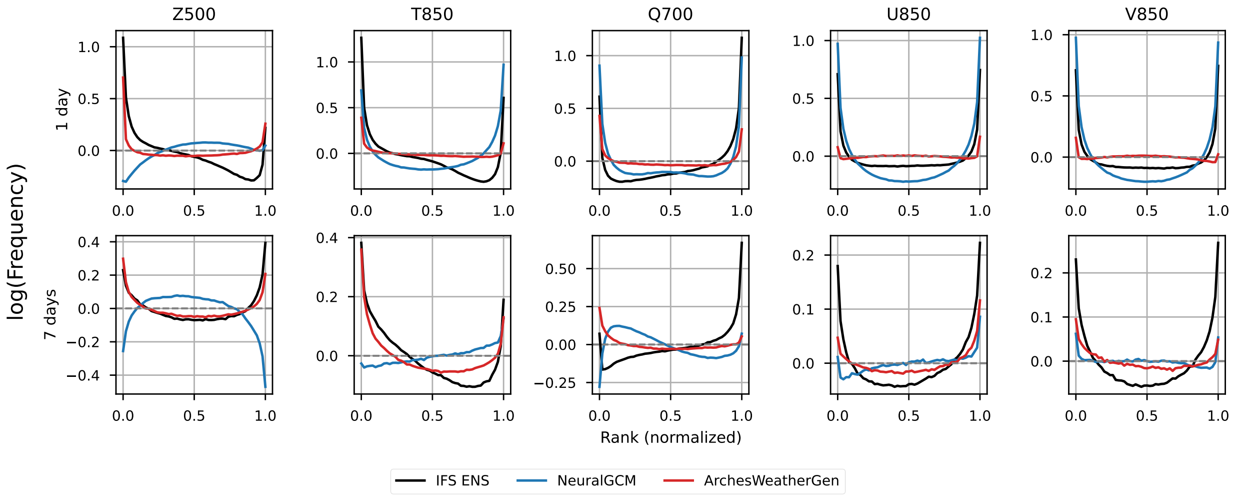

Figure S5: **Rank Histogram of ArchesWeatherGen compared to NeuralGCM and IFS ENS.** The rank histograms of ArchesWeatherGen are much flatter than the other methods at 1 day lead time. At 7 days lead time, its rank histograms are better than NeuralGCM for *Z500* and *Q700* but slightly worse for the other variables due to remaining under-dispersiveness.

Impact of OOD fine-tuning and noise scaling As explained in Section , overfitting of the underlying deterministic model results in test residuals that are slightly "out-of-distribution" compared to residuals on the train set, and in particular have a higher norm than on the train set, which results in under-dispersion. We now evaluate the effectiveness of our strategies to mitigate this problem: fine-tuning the generative model on year 2019 (on which the deterministic model has not been trained) and scaling the variance of the noise given as input to the generative model.

In Figure S6, we first see that our flow matching model greatly improves upon the DDPM variant for all ensemble metrics but notably (and perhaps unexpectedly) for spread-skill ratio: at 24h, the DDPM version has a spread-skill ratio of 0.68, compared to 0.85 for the basic flow matching version without OOD fine-tuning or noise scaling. Building upon this base version, we add OOD fine-tuning which improves all metrics and has a better 24h spread-skill ratio of 0.9. Finally, with

noise scaling on top, the ensemble metrics are not very different, but the spread-skill ratio is improved to around 0.96 and increases to 0.98 at longer lead times. Overall, this experiment shows that fine-tuning helps combat distribution mismatch and improve all ensemble metrics. On the other hand, noise scaling has no effect on RMSE, CRPS or BrierScore, but helps to improve the dispersiveness of our model.

We also tried a more naive way of increasing the dispersion of the model, which is to rescale the output of the residual generative model. Although this was effective in adjusting the spread-skill ratio, the other ensemble metrics (and notably CRPS) were degraded, which is expected since the probability distribution of residuals with a higher norm is a priori different from the unconditional distribution of residuals. On the other hand, a higher-norm input noise is guided by our flow matching model towards the true probability distribution of higher-norm residuals, as evidenced by the higher dispersion obtained with similar or slightly higher CRPS scores.

In figure S7, we report the CRPS scores of various methods separately for each headline variable in WeatherBench. We can see that the ranking of methods is the same for all variables, with ArchesWeatherGen having the best scores and ArchesWeather-DDPM the lowest.

In figure S8, we report the spread-skill ratio of our methods separately for each headline variable in WeatherBench. Again, we can see that the qualitative behavior is the same for all variables. Using noise scaling helps to reach a spread-skill ratio close to 1 for all lead times, except for 2m-temperature.

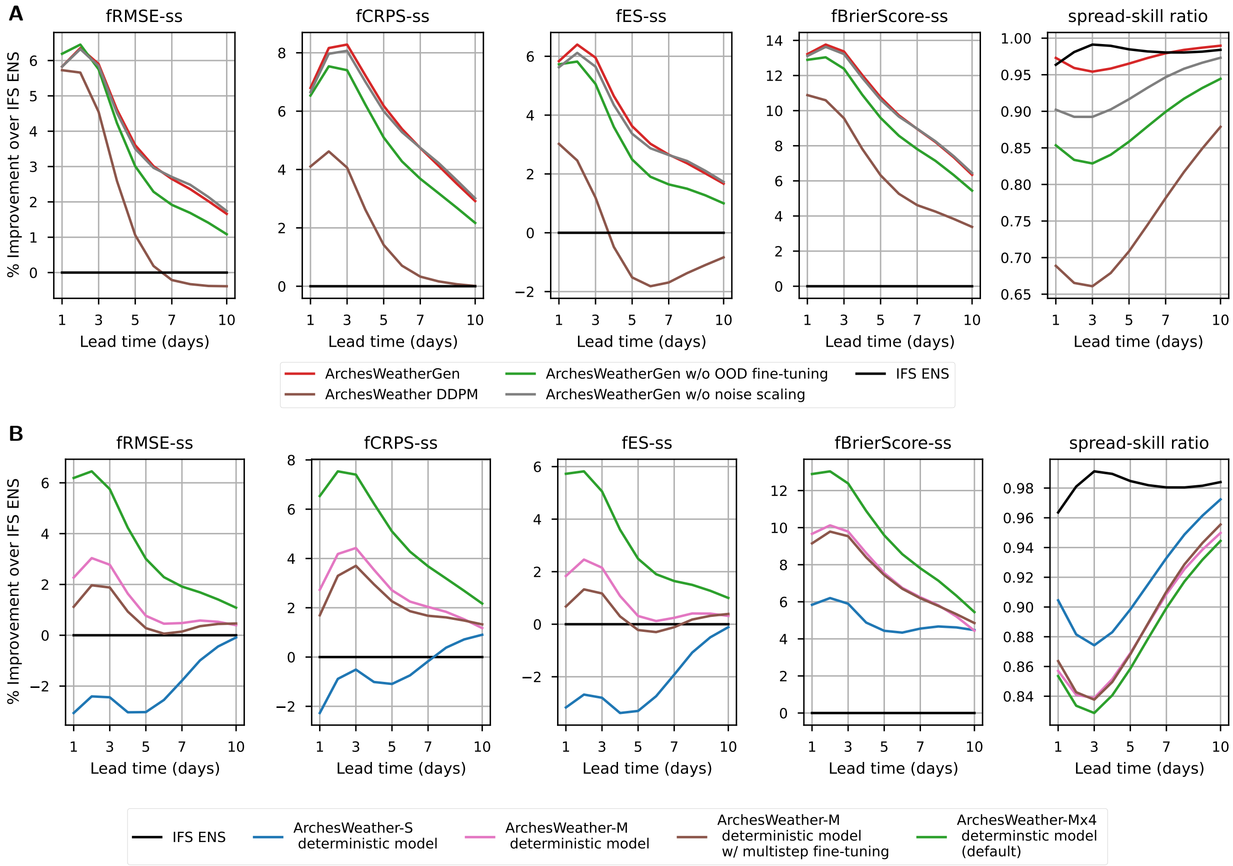

Figure S6: **Ablation study of key design choices of ArchesWeatherGen. A:** Ablation of our strategies to improve ensemble metrics and dispersion of residual generative models. OOD fine-tuning helps to improve all ensemble metrics, while noise scaling only reduces under-dispersion. **B:** Impact of the choice of deterministic model used in residual modeling on ensemble metrics. Better deterministic models improve all metrics, especially at shorter lead times.

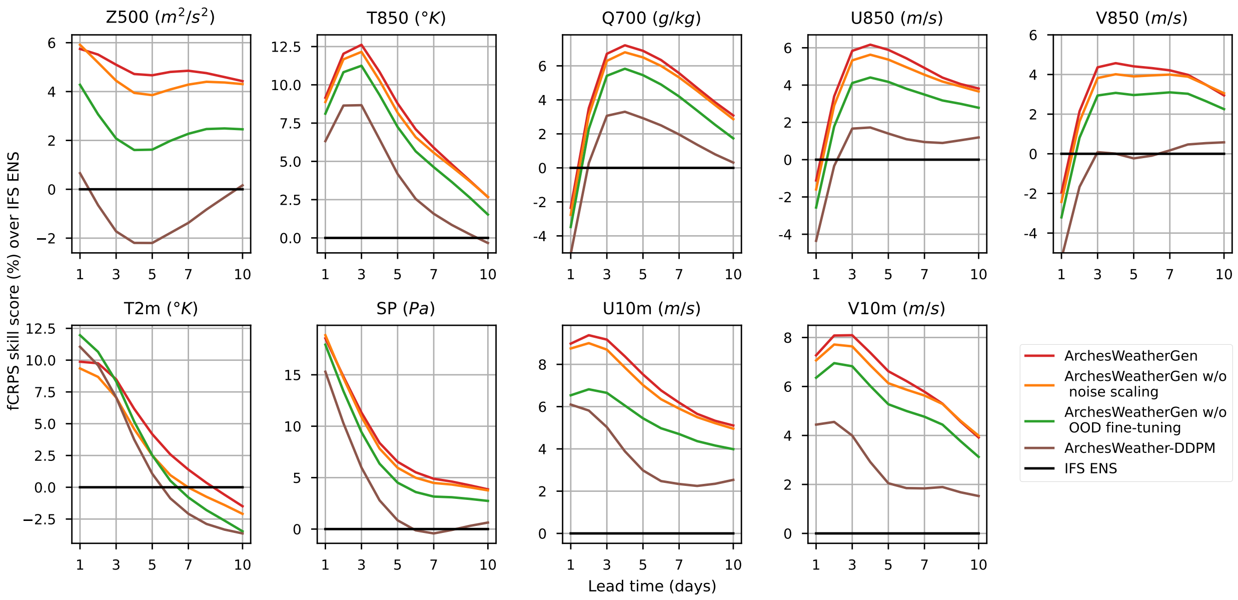

Figure S7: **fCRPS scores of ArchesWeatherGen variants per headline variable.** We can see similar trends for all variables.

Impact of deterministic model Our residual generative models require a choice of deterministic model to compute the residuals on which it is trained. In Figure S6, we evaluate the impact of this design choice on the performance of our flow matching models. We use four different deterministic models: ArchesWeather-S, ArchesWeather-M, ArchesWeather-M with multi-step fine-tuning, and ArchesWeather-Mx4, the latter being the one used in ArchesWeatherGen.

We can see that the choice of underlying deterministic model has a big impact on ensemble metrics, with bigger deterministic models yielding better residual generative models. The best results are with the ArchesWeather-Mx4 as underlying deterministic model, substantially better than with a single ArchesWeather-M model, demonstrating the importance of removing neural network initialization bias with ensembling. Using better deterministic models than ArchesWeather-Mx4 could potentially bring further gains. One small downside of using larger

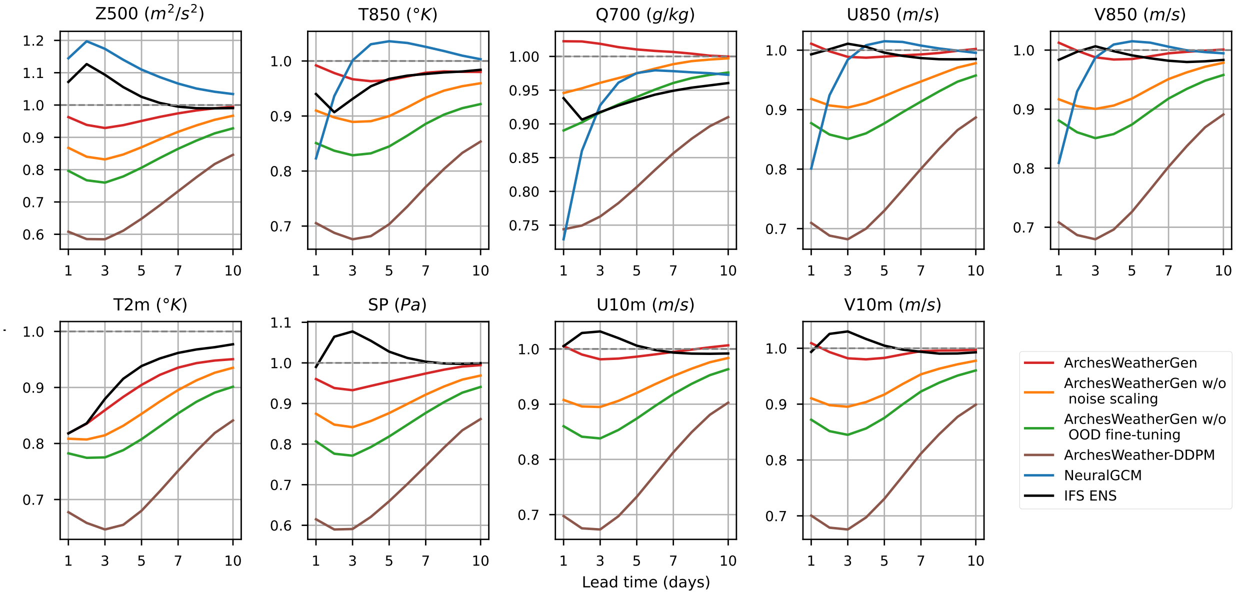

Figure S8: **Spread-skill ratios of our methods with different variables.** Using OOD and noise scaling helps to recover correct dispersiveness across lead times.

deterministic models is that the spread-skill ratio is slightly decreased, which is another manifestation of overfitting, but we have proposed solutions to handle this problem. Finally, an interesting observation is to compare a deterministic model trained with next-step prediction only versus one that has been fine-tuned on auto-regressive rollouts. Using the fine-tuned model seems to decrease performance of our generative model, probably because it is slightly worse for next-step prediction compared to the model without multi-step fine-tuning.

5 Additional visualizations

5.1 Samples generated on Hurricane Teddy

In figure S9 we can see 25 generations of a 7-days rollout of ArchesWeatherGen. Of these, we can see that the hurricane structure dissipated in three of them, with

the rest having different degrees of intensification. The trajectories are physically realizable, and most of them follow trajectories that would lead to accurate warnings of an intensification of the hurricane, giving ample warning time.

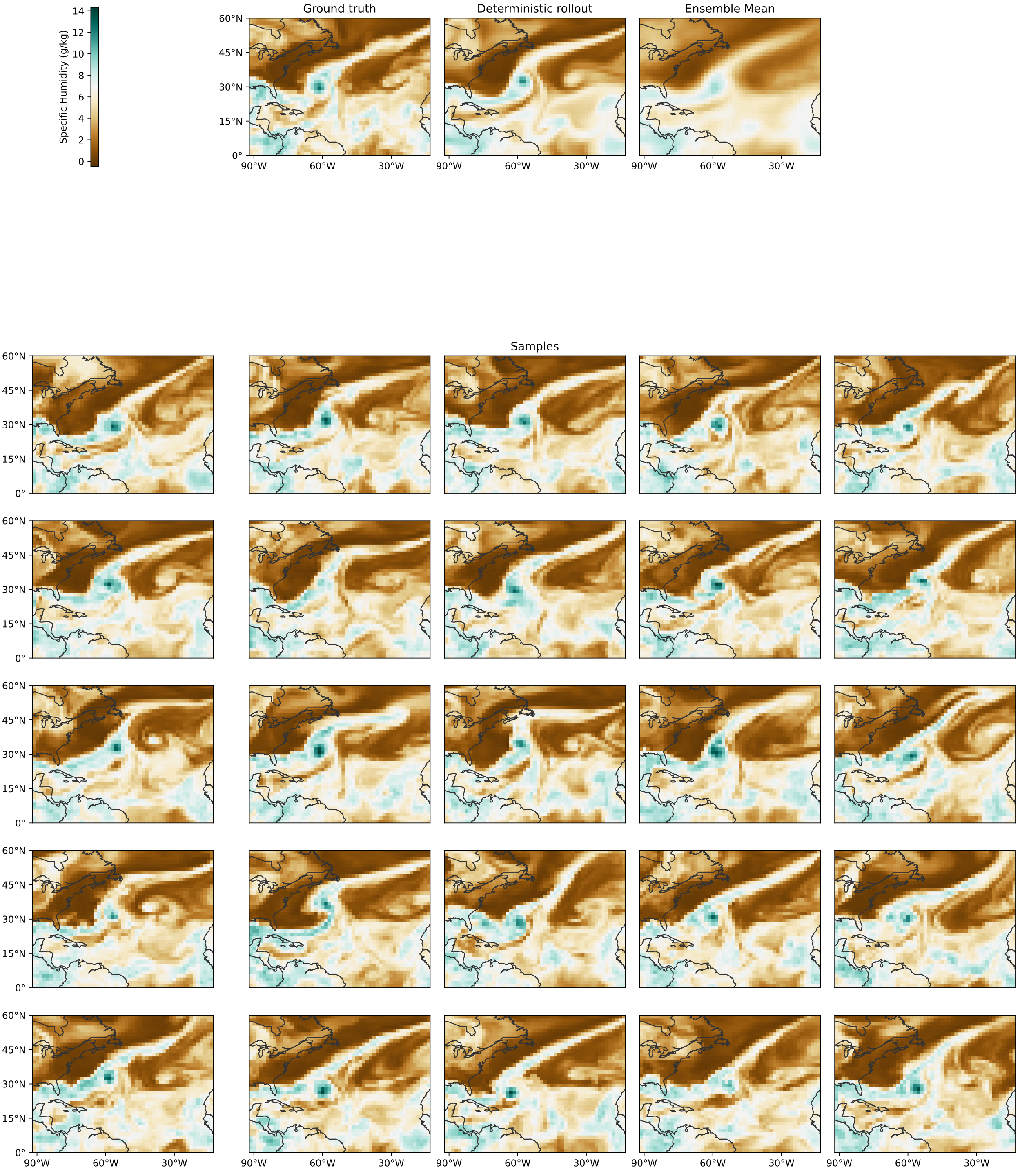

Figure S9: 25-member 7-day forecast generated by ArchesWeatherGen on Hurricane Teddy, initialized September 14th, 2020.

REFERENCES

1. H. Hersbach, B. Bell, P. Berrisford, S. Hirahara, A. Horányi, J. Muñoz-Sabater, J. Nicolas, C. Peubey, R. Radu, D. Schepers, A. Simmons, C. Soci, S. Abdalla, X. Abellan, G. Balsamo, P. Bechtold, G. Biavati, J. Bidlot, M. Bonavita, G. De Chiara, P. Dahlgren, D. Dee, M. Diamantakis, R. Dragani, J. Flemming, R. Forbes, M. Fuentes, A. Geer, L. Haimberger, S. Healy, R. J. Hogan, E. Hólm, M. Janisková, S. Keeley, P. Laloyaux, P. Lopez, C. Lupu, G. Radnoti, P. de Rosnay, I. Rozum, F. Vamborg, S. Villaume, J.-N. Thépaut, The ERA5 global reanalysis. *Q. J. Roy. Meteorol. Soc.* **146**, 1999–2049 (2020).

2. K. Bi, L. Xie, H. Zhang, X. Chen, X. Gu, Q. Tian, Accurate medium-range global weather forecasting with 3D neural networks. *Nature* **619**, 533–538 (2023).

3. J. Pathak, S. Subramanian, P. Harrington, S. Raja, A. Chattopadhyay, M. Mardani, T. Kurth, D. Hall, Z. Li, K. Azizzadenesheli, P. Hassanzadeh, K. Kashinath, A. Anandkumar, Fourcastnet: A global data-driven high-resolution weather model using adaptive fourier neural operators. arXiv:2202.11214 [physics.ao-ph] (2022).

4. R. Lam, A. Sanchez-Gonzalez, M. Willson, P. Wirnsberger, M. Fortunato, F. Alet, S. Ravuri, T. Ewalds, Z. Eaton-Rosen, W. Hu, A. Merose, S. Hoyer, G. Holland, O. Vinyals, J. Stott, A. Pritzel, S. Mohamed, P. Battaglia, Learning skillful medium-range global weather forecasting. *Science* **382**, 1416–1421 (2023).

5. L. Chen, X. Zhong, F. Zhang, Y. Cheng, Y. Xu, Y. Qi, H. Li, FuXi: A cascade machine learning forecasting system for 15-day global weather forecast. *Science* **6**, 190 (2023).

6. T. Nguyen, R. Shah, H. Bansal, T. Arcomano, R. Maulik, R. Kotamarthi, I. Foster, S. Madireddy, A. Grover, Scaling transformer neural networks for skillful and reliable medium-range weather forecasting. *Adv. Neural Inf. Process. Syst.* **37**, 68740–68771 (2024).

7. E. Guo, M. Ahmed, Y. Sun, R. Mahendru, R. Yang, H. Cook, T. Leeuwenburg, B. Evans, FourCastNeXt: Improving fourcastnet training with limited compute. arXiv:2401.05584 [cs. CV] (2024).

8. D. Kochkov, J. Yuval, I. Langmore, P. Norgaard, J. Smith, G. Mooers, M. Klöwer, J. Lottes, S. Rasp, P. Düben, S. Hatfield, P. Battaglia, A. Sanchez-Gonzalez, M. Willson, M. P. Brenner, S. Hoyer, Neural general circulation models for weather and climate. *Nature* **632**, 1060–1066 (2024).

9. C. Lessig, I. Luise, B. Gong, M. Langguth, S. Stadler, M. Schultz, AtmoRep: A stochastic model of atmosphere dynamics using large scale representation learning. arXiv:2308.13280 [physics.ao-ph] (2023).

10. A. Dosovitskiy, L. Beyer, A. Kolesnikov, D. Weissenborn, X. Zhai, T. Unterthiner, M. Dehghani, M. Minderer, G. Heigold, S. Gelly, J. Uszkoreit, N. Houlsby, An image is worth 16x16 words: Transformers for image recognition at scale (2021), https://openreview.net/forum?id=YicbFdNTTy.

11. M. Bonavita, On some limitations of current machine learning weather prediction models. *Geophys. Res. Lett.* **51**, e2023GL107377 (2024).

12. A. Ramesh, M. Pavlov, G. Goh, S. Gray, C. Voss, A. Radford, M. Chen, I. Sutskever, "Zero-shot text-to-image generation," in *International Conference on Machine Learning* (PMLR, 2021), pp. 8821–8831.

13. P. Esser, R. Rombach, B. Ommer, "Taming transformers for high-resolution image synthesis," in *Proceedings of the IEEE/CVF Conference on Computer Vision and Pattern Recognition* (IEEE, 2021), pp. 12,873–12,883.

14. C. Saharia, W. Chan, S. Saxena, L. Li, J. Whang, E. Denton, S. K. S. Ghasemipour, B. K. Ayan, S. S. Mahdavi, R. G. Lopes, T. Salimans, J. Ho, D. J. Fleet, M. Norouzi, Photorealistic text-to-image diffusion models with deep language understanding. *Adv. Neural Inf. Process. Syst.* **35**, 36479–36494 (2022).

15. R. Rombach, A. Blattmann, D. Lorenz, P. Esser, B. Ommer, "High-resolution image synthesis with latent diffusion models," in *Proceedings of the IEEE/CVF Conference on Computer Vision and Pattern Recognition* (IEEE, 2022), pp. 10,684–10,695.

16. I. Price, A. Sanchez-Gonzalez, F. Alet, T. R. Andersson, A. El-Kadi, D. Masters, T. Ewalds, J. Stott, S. Mohamed, P. Battaglia, R. Lam, M. Willson, Probabilistic weather forecasting with machine learning. *Nature* **637**, 84–90 (2025).

17. S. Shang, Z. Shan, G. Liu, L. Wang, X. Wang, Z. Zhang, J. Zhang, "Resdiff: Combining cnn and diffusion model for image super-resolution," in *Proceedings of the AAAI Conference on Artificial Intelligence* (AAAI Press, 2024), vol. 38, pp. 8975–8983.

18. M. Mardani, N. Brenowitz, Y. Cohen, J. Pathak, C.-Y. Chen, C.-C. Liu, A. Vahdat, M. A. Nabian, T. Ge, A. Subramaniam, K. Kashinath, J. Kautz, M. Pritchard, Residual corrective diffusion modeling for km-scale atmospheric downscaling. *Commun. Earth Environ.* **6**, 124 (2025).

19. J. Ho, A. Jain, P. Abbeel, Denoising diffusion probabilistic models. *Adv. Neural. Inf. Process. Syst.* **33**, 6840–6851 (2020).

20. T. Karras, M. Aittala, T. Aila, S. Laine, Elucidating the design space of diffusion-based generative models. *Adv. Neural. Inf. Process. Syst.* **35**, 26565–26577 (2022).

21. P. Esser, S. Kulal, A. Blattmann, R. Entezari, J. Müller, H. Saini, Y. Levi, D. Lorenz, A. Sauer, F. Boesel, D. Podell, T. Dockhorn, Z. English, K. Lacey, A. Goodwin, Y. Marek, R. Rombach, "Scaling rectified flow transformers for high-resolution image synthesis," in *ICML'24: Proceedings of the 41st International Conference on Machine Learning* (2024), pp. 12,606–12,633.

22. Y. Lipman, R. T. Chen, H. Ben-Hamu, M. Nickel, M. Le, "Flow matching for generative modeling," in *11th International Conference on Learning Representations* (2023), pp. 28.

23. J. Song, C. Meng, S. Ermon, "Denoising diffusion implicit models," *International Conference on Learning Representations* (2020).

24. A. Vaswani, N. Shazeer, N. Parmar, J. Uszkoreit, L. Jones, A. N. Gomez, L. Kaiser, I. Polosukhin, Attention is all you need. *Advances in Neural Information Processing Systems* (2017), vol 30, pp. 5998–6008.

25. Z. Liu, H. Hu, Y. Lin, Z. Yao, Z. Xie, Y. Wei, J. Ning, Y. Cao, Z. Zhang, L. Dong, F. Wei, B. Guo, "Swin transformer v2: Scaling up capacity and resolution," in *Proceedings of the IEEE/CVF Conference on Computer Vision and Pattern Recognition* (IEEE, 2022), pp. 12,009–12,019.

26. L. Chen, F. Du, Y. Hu, Z. Wang, F. Wang, "Swinrdm: Integrate swinrnn with diffusion model towards high-resolution and high-quality weather forecasting," in *Proceedings of the AAAI Conference on Artificial Intelligence* (AAAI Press, 2023), vol. 37, pp. 322–330.

27. X. Zhong, L. Chen, J. Liu, C. Lin, Y. Qi, H. Li, FuXi-Extreme: Improving extreme rainfall and wind forecasts with diffusion model. *Sci. China Earth Sci.* **67**, 3696–3708 (2024).

28. L. Li, R. Carver, I. Lopez-Gomez, F. Sha, J. Anderson, Generative emulation of weather forecast ensembles with diffusion models. *Sci. Adv.* **10**, eadk4489 (2024).

29. C. Brochet, G. Moldovan, L. Raynaud, M. Plu, "Using state-of-the-art generative neural networks for high-resolution NWP ensemble emulation" (Tech. Rep., Copernicus Meetings, 2023).

30. P. Srivastava, R. Yang, G. Kerrigan, G. Dresdner, J. McGibbon, C. Bretherton, S. Mandt, Probabilistic precipitation downscaling with optical flow-guided diffusion. arXiv:2312.06071v1 [cs.CV] (2023).

31. R. A. Watt, L. A. Mansfield, Generative diffusion-based downscaling for climate. arXiv:2404.17752 [physics.ao-ph] (2024).

32. E. Tomasi, G. Franch, M. Cristoforetti, Can AI be enabled to perform dynamical downscaling? A latent diffusion model to mimic kilometer-scale COSMO5.0_CLM9 simulations. *EGUsphere* **18**, 2051–2078 (2024).

33. Q. Han, X. Jiang, Y. Zhao, X. Wang, Z. Li, R. Zhang, Diffusion-model-based downscaling of observed sea surface height over the kuroshio extension since 2000. *Atmos.* **16**, 570 (2025).

34. I. Lopez-Gomez, Z. Y. Wan, L. Zepeda-Núñez, T. Schneider, J. Anderson, F. Sha, Dynamical-generative downscaling of climate model ensembles. *Proc. Natl. Acad. Sci. U.S.A.* **122**, e2420288122 (2025).

35. D. Yu, X. Li, Y. Ye, B. Zhang, C. Luo, K. Dai, R. Wang, X. Chen, "Diffcast: A unified framework via residual diffusion for precipitation nowcasting," in *Proceedings of the IEEE/CVF Conference on Computer Vision and Pattern Recognition* (IEEE, 2024), pp. 27758–27767.

36. Z. Zhao, X. Dong, Y. Wang, C. Hu, Advancing realistic precipitation nowcasting with a spatiotemporal transformer-based denoising diffusion model. *IEEE Trans. Geosci. Remote Sens.* **62**, 1–15 (2024).

37. J. Gong, L. Bai, P. Ye, W. Xu, N. Liu, J. Dai, X. Yang, W. Ouyang, "CasCast: Skillful high-resolution precipitation nowcasting via cascaded modelling," in *International Conference on Machine Learning* (PMLR, 2024).

38. H. Addison, E. Kendon, S. Ravuri, L. Aitchison, P. A. Watson, Machine learning emulation of precipitation from km-scale regional climate simulations using a diffusion model. arXiv:2407.14158 [physics.ao-ph] (2024).

39. C. Guilloteau, G. Kerrigan, K. Nelson, G. Migliorini, P. Smyth, R. Li, E. Foufoula-Georgiou, A generative diffusion model for probabilistic ensembles of precipitation maps conditioned on multisensor satellite observations. *IEEE Trans. Geosci. Remote Sens.* **63**, 1–15 (2025).

40. P. Nath, P. Shukla, S. Wang, C. Quilodrán-Casas, Forecasting tropical cyclones with cascaded diffusion models. arXiv:2310.01690 [physics.ao-ph] (2023).

41. C. Huang, P. Mu, C. Bai, P. A. Watson, "TCP-diffusion: A multi-modal diffusion model for global tropical cyclone precipitation forecasting with change awareness," *International Conference on Machine Learning* (PMLR, 2024); https://openreview.net/forum?id=9cOtYlD5UA.

42. T. S. Finn, C. Durand, A. Farchi, M. Bocquet, J. Brajard, Towards diffusion models for large-scale sea-ice modelling. arXiv:2406.18417 [cs.LG] (2024).

43. L. Huang, L. Gianinazzi, Y. Yu, P. D. Dueben, T. Hoefler, "DiffDA: A diffusion model for weather-scale data assimilation," *International Conference on Machine Learning* (PMLR, 2024); https://openreview.net/forum?id=vhMq3eAB34.

44. F. Rozet, G. Louppe, Score-based data assimilation. *Adv. Neural Inf. Process Syst.* **36**, 40521–40541 (2023).

45. P. Manshausen, Y. Cohen, J. Pathak, M. Pritchard, P. Garg, M. Mardani, K. Kashinath, S. Byrne, N. Brenowitz, Generative data assimilation of sparse weather station observations at kilometer scales. arXiv:2406.16947 [cs.LG] (2024).

46. C. Esteves, J.-J. Slotine, A. Makadia, Scaling spherical CNNs. arXiv:2306.05420 [cs.LG] (2023).

47. J. A. Weyn, D. R. Durran, R. Caruana, Improving data-driven global weather prediction using deep convolutional neural networks on a cubed sphere. *J. Adv. Model. Earth Syst.* **12**, e2020MS002109 (2020).

48. K. Pandey, J. Pathak, Y. Xu, S. Mandt, M. Pritchard, A. Vahdat, M. Mardani, "Heavy-tailed diffusion models," in *International Conference on Learning Representations* (2024); https://openreview.net/forum?id=tozlOEN4qp.

49. L. Isaksen, M. Bonavita, R. Buizza, M. Fisher, J. Haseler, M. Leutbecher, L. Raynaud, *Ensemble of Data Assimilations at ECMWF* (ECMWF, 2010).

50. S. Rasp, S. Hoyer, A. Merose, I. Langmore, P. Battaglia, T. Russell, A. Sanchez-Gonzalez, V. Yang, R. Carver, S. Agrawal, M. Chantry, Z. Ben Bouallegue, P. Dueben, C. Bromberg, J. Sisk, L. Barrington, A. Bell, F. Sha, WeatherBench 2: A benchmark for the next generation of data-driven global weather models. *J. Adv. Model. Earth Syst.* **16**, e2023MS004019 (2024).

51. Z. Liu, Y. Lin, Y. Cao, H. Hu, Y. Wei, Z. Zhang, S. Lin, B. Guo, "Swin transformer: Hierarchical vision transformer using shifted windows," in *Proceedings of the IEEE/CVF International Conference on Computer Vision* (IEEE, 2021), pp. 10,012–10,022.

52. J. Ho, N. Kalchbrenner, D. Weissenborn, T. Salimans, Axial attention in multidimensional transformers. arXiv:1912.12180 [cs.CV] (2019).

53. M. Andrychowicz, L. Espeholt, D. Li, S. Merchant, A. Merose, F. Zyda, S. Agrawal, N. Kalchbrenner, Deep learning for day forecasts from sparse observations. arXiv:2306.06079 [physics.ao-ph] (2023).

54. A. Aitken, C. Ledig, L. Theis, J. Caballero, Z. Wang, W. Shi, Checkerboard artifact free sub-pixel convolution: A note on sub-pixel convolution, resize convolution and convolution resize. arXiv:1707.02937 [cs.CV] (2017).

55. N. Shazeer, Glu variants improve transformer. arXiv:2002.05202 [cs.LG] (2020).

56. H. Touvron, T. Lavril, G. Izacard, X. Martinet, M.-A. Lachaux, T. Lacroix, B. Rozière, N. Goyal, E. Hambro, F. Azhar, A. Rodriguez, A. Joulin, E. Grave, G. Lample, Llama: Open and efficient foundation language models. arXiv:2302.13971 [cs.CL] (2023).

57. E. Perez, F. Strub, H. De Vries, V. Dumoulin, A. Courville, "Film: Visual reasoning with a general conditioning layer," in *Proceedings of the AAAI Conference on Artificial Intelligence* (AAAI Press, 2018), vol. 32.

58. A. Krogh, J. Vedelsby, Neural network ensembles, cross validation, and active learning. *Adv. Neural Inf. Process. Syst.* **7**, 231–238 (1994).

59. Z. Ben Bouallegue, Accuracy versus activity. ECMWF website (2024), 10.21957/8b50609a0f; https://www.ecmwf.int/en/about/media-centre/aifs-blog/2024/accuracy-versus-activity.

60. J. Sohl-Dickstein, E. Weiss, N. Maheswaranathan, S. Ganguli, "Deep unsupervised learning using nonequilibrium thermodynamics," in *International Conference on Machine Learning (*PMLR, 2015), pp. 2256–2265.

61. A. Q. Nichol, P. Dhariwal, "Improved denoising diffusion probabilistic models," in *International Conference on Machine Learning (*PMLR, 2021), pp. 8162–8171.

62. L. Weng, What are diffusion models? (2021), p. 21, https://lilianweng.github.io/.

63. H. Yu, L. Shen, J. Huang, H. Li, F. Zhao, Unmasking Bias in Diffusion Model Training. arXiv preprint arXiv:2310.08442 (2023).

64. P. Dhariwal, A. Nichol, Diffusion models beat gans on image synthesis. *Adv. Neural Inf. Process. Syst.* **34**, 8780–8794 (2021).

65. G. Couairon, J. Verbeek, H. Schwenk, M. Cord, "DiffEdit: Diffusion-based semantic image editing with mask guidance," in *International Conference on Learning Representations* (2023), https://openreview.net/forum?id=3lge0p5o-M-.

66. C. Ferro, Fair scores for ensemble forecasts. *Q. J. Roy. Meteorol. Soc.* **140**, 1917–1923 (2014).

67. T. Gneiting, A. E. Raftery, Strictly proper scoring rules, prediction, and estimation. *J. Am. Stat. Assoc.* **102**, 359–378 (2007).

68. M. S. Roulston, Performance targets and the Brier score. *Met. Apps.* **14**, 185–194 (2007).

69. V. Fortin, M. Abaza, F. Anctil, R. Turcotte, Why should ensemble spread match the RMSE of the ensemble mean? *J. Hydrometeorol.* **15**, 1708–1713 (2014).

70. T. M. Hamill, Interpretation of rank histograms for verifying ensemble forecasts. *Mon. Wea. Rev.* **129**, 550–560 (2001).

71. H. Hersbach, B. Bell, P. Berrisford, G. Biavati, A. Horányi, J. Muñoz Sabater, J. Nicolas, C. Peubey, R. Radu, I. Rozum, D. Schepers, A. Simmons, C. Soci, D. Dee, J.-N. Thépaut, ERA5 hourly data on single levels from 1940 to present. *Copernicus Climate Change Service (C3S) Climate Data Store (CDS)* (2023), accessed on 19 December 2024; 10.24381/cds.adbb2d47.

72. G. Deepmind, WeatherNext, a family of AI models from Google DeepMind and Google Research, produces state-of-the-art weather forecasts. (2025), https://deepmind.google/science/weathernext/#access-weathernext.

73. G. Couairon, R. Singh, GeoArches (2024); https://github.com/INRIA/geoarches.

74. D. P. Kingma, J. Ba, Adam: A method for stochastic optimization. arXiv:1412.6980 [cs.LG] (2014).

75. L. AI, A100 vs V100 Deep Learning Benchmarks (2021); https://lambda.ai/blog/nvidia-a100-vs-v100-benchmarks.

76. N. P. Jouppi, G. Kurian, S. Li, P. Ma, R. Nagarajan, L. Nai, N. Patil, S. Subramanian, A. Swing, B. Towles, C. Young, X. Zhou, Z. Zhou, D. Patterson, "Tpu v4: An optically reconfigurable supercomputer for machine learning with hardware support for embeddings," in *Proceedings of the 50th Annual International Symposium on Computer Architecture* (IEEE / ACM, 2023), pp. 1–14.

77. G. C. Blog, Enabling next-generation AI workloads: Announcing TPU v5p and AI Hypercomputer (2023), https://cloud.google.com/blog/products/ai-machine-learning/introducing-cloud-tpu-v5p-and-ai-hypercomputer.

78. C. A. T. Ferro, Comparing probabilistic forecasting systems with the brier score. *Weather Forecast.* **22**, 1076–1088 (2007).

79. P. Bauer, A. Thorpe, G. Brunet, The quiet revolution of numerical weather prediction. *Nature* **525**, 47–55 (2015).

80. O. Talagrand, "Evaluation of probabilistic prediction systems," in *Workshop Proceedings "Workshop on Predictability," 20–22 October 1997*, (ECMWF, Reading, UK 1999).
